# Pathological manifestations in lymphatic filariasis correlate with lack of inhibitory properties of IgG4 antibodies on IgE-activated granulocytes

**DOI:** 10.1371/journal.pntd.0005777

**Published:** 2017-07-24

**Authors:** Ulrich F. Prodjinotho, Charlotte von Horn, Alex Y. Debrah, Linda Batsa Debrah, Anna Albers, Laura E. Layland, Achim Hoerauf, Tomabu Adjobimey

**Affiliations:** 1 Institute of Medical Microbiology, Immunology and Parasitology (IMMIP), University Hospital Bonn, Bonn, Germany; 2 Kumasi Centre for Collaborative Research in Tropical Medicine (KCCR), Kwame Nkrumah University of Science and Technology, Kumasi, Ghana; 3 Faculty of Allied Health Sciences and School of Medical Sciences, Kwame Nkrumah University of Science and Technology, Kumasi, Ghana; 4 Department of Clinical Microbiology, Kwame Nkrumah University of Science and Technology, Kumasi, Ghana; 5 Bonn-Cologne Site, German Center for Infectious Disease Research (DZIF), Bonn, Germany; 6 Faculté des Sciences et Techniques (FAST), Université d’Abomey-Calavi, Abomey-Calavi, Bénin; Uniformed Services University of the Health Sciences, UNITED STATES

## Abstract

Helminth parasites are known to be efficient modulators of their host’s immune system. To guarantee their own survival, they induce alongside the classical Th2 a strong regulatory response with high levels of anti-inflammatory cytokines and elevated plasma levels of IgG4. This particular antibody was shown in different models to exhibit immunosuppressive properties. How IgG4 affects the etiopathology of lymphatic filariasis (LF) is however not well characterized. Here we investigate the impact of plasma and affinity-purified IgG/IgG4 fractions from endemic normals (EN) and LF infected pathology patients (CP), asymptomatic microfilaraemic (Mf+) and amicrofilaraemic (Mf-) individuals on IgE/IL3 activated granulocytes. The activation and degranulation states were investigated by monitoring the expression of CD63/HLADR and the release of granule contents (neutrophil elastase (NE), eosinophil cationic protein (ECP) and histamine) respectively by flow cytometry and ELISA. We could show that the activation of granulocytes was inhibited in the presence of plasma from EN and Mf+ individuals whereas those of Mf- and CP presented no effect. This inhibitory capacity was impaired upon depletion of IgG in Mf+ individuals but persisted in IgG-depleted plasma from EN, where it strongly correlated with the expression of IgA. In addition, IgA-depleted fractions failed to suppress granulocyte activation. Strikingly, affinity-purified IgG4 antibodies from EN, Mf+ and Mf- individuals bound granulocytes and inhibited activation and the release of ECP, NE and histamine. In contrast, IgG4 from CP could not bind granulocytes and presented no suppressive capacity. Reduction of both the affinity to, and the suppressive properties of anti-inflammatory IgG4 on granulocytes was reached only when FcγRI and II were blocked simultaneously. These data indicate that IgG4 antibodies from Mf+, Mf- and EN, in contrast to those of CP, natively exhibit FcγRI/II-dependent suppressive properties on granulocytes. Our findings suggest that quantitative and qualitative alterations in IgG4 molecules are associated with the different clinical phenotypes in LF endemic regions.

## Author summary

Lymphatic Filariasis, also known as elephantiasis, infects an estimated 39 million people in 73 tropical and sub-tropical countries. The most severe clinical manifestations of the disease include swelling of the scrotal area and lower limbs (hydrocele and lymphedema). It is well admitted that host immune reactivity plays a critical role in the pathogenesis of the disease. Previous investigations have linked the non-cytolytic antibody IgG4 to the hyporesponsive states in filarial infections. However, few data exist on how this antibody is involved in the pathogenesis of human filariasis. Here we investigated the role of this antibody in disease pathogenesis by comparing the effect of plasma, IgG and IgG4 fractions from the four clinical categories of individuals; chronic pathology individuals (CP), asymptomatic microfilaria positive (Mf+) and negative (Mf-) and uninfected endemic normal individuals (EN) on activated granulocytes. We could show that granulocyte activation was significantly inhibited in the presence of plasma from EN and Mf+ and that, affinity-purified IgG4 antibodies from EN, Mf+ and Mf- individuals inhibited granulocyte activation in a dose-dependent manner via the immune receptors FcγRI and FcγRII. Our data also reveal significant functional differences between IgG4 molecules from EN, Mf+, Mf- and CP.

## Introduction

Lymphatic filariasis (LF) also known as elephantiasis is a potentially disabling and disfiguring disease caused in human by vector-borne nematodes *Wuchereria bancrofti*, *Brugia malayi* and *Brugia timori* [1]. The disease has significant social and economic consequences for affected individuals as well as for their families and communities [2]. The current strategy to control the infection is based on mass administration (MDA) of diethylcarbamazine or ivermectin combined to albendazole [2]. After 13 years of the MDA programme, recent estimations in 2015 indicate that 38.47 million LF cases remain [3]. In endemic regions, exposure to the infection leads to different clinical phenotypes. The first group includes putatively immune individuals or endemic normals (EN) who remain infection and disease-free despite continuous exposition to mosquito-transmitted infective larvae (L3) [4]. The second group is defined by a hyper-reactive phenotype and is characterized by chronic lymphatic pathologies (CP) such as lymphedema or elephantiasis. The patients elicit a strong T helper (Th)1 and Th17 immune response that eliminate the microfilarial stage [5] while inducing the production of angiogenic factors like VEGF known to be associated with the development of filarial lymphedema [6]. This severe clinical profile is characterized by high antigen-specific immunoglobulin (Ig)E and low IgG4 [5] [7,8]. The third group includes asymptomatic individuals with latent infection who are free of microfilaria (Mf-) but are positive for circulating filarial antigens (CFA) [9]. The last clinical phenotype includes the majority of infected individuals and is associated with an hyporesponsive immune profile. In this group, individuals present few visible clinical manifestations despite large numbers of circulating microfilariae (Mf+) [10–12]. Subjects from this group commonly present a modified Th2 immune profile with a strong parasite-specific immunoregulatory arm allowing both the presence of adult worms and microfilariae. This modified Th2 response is associated with increased numbers of regulatory cells (Tregs) and alternatively activated macrophages as well as with the secretion of anti-inflammatory cytokines such as IL-10 and TGF-β. This predominantly immunosuppressed environment is associated with elevated levels of antigen specific IgG4 and is directly linked with parasite survival [4,10,13].

These clinical phenotypes strongly correlate with specific antibody isotypes produced in response to the infection. IgG4 antibodies, for example, correlate with the hyporesponsive state observed in Mf+ individuals whereas IgE, IgG1 and IgG3 correlate with CP [7,10,14–16]. IgG4 is structurally and functionally different from its co-class members [17–19]. While IgG1, IgG2 and IgG3 can fix and activate complement, IgG4 has no affinity for the complement and cannot induce antibody-dependent cell mediated cytotoxicity (ADCC). In addition, IgG4 was shown to inhibit antibody dependent complement activation [20] and to compete with IgE for fixation sites on mast cells and eosinophils [21,22]. In contrast, IgE induces mast cell and basophil degranulation [23–25]. These anti-inflammatory properties of IgG4 antibodies were associated with its unique ability to undergo Fab-arm exchange (FAE); resulting in the creation of bispecific, functionally monovalent antibodies [26,27]. However, the role played by filaria-induced antibodies in disease manifestations in LF is still not well understood. No data currently exist on how IgG4 antibodies participate in the modulation of the pathophysiology of filarial infections.

Granulocytes (eosinophils, neutrophils, and basophils) are key effector cells at the frontline against infections with filarial worms [28,29]. During helminth infection, granulocytes are rapidly activated and recruited to sites of infection where they are key producers of Th2 cytokines such as IL-4 and IL-13 [28,30,31]. They also produce “alarmins” which are constitutively available endogenous molecules that are released upon activation and act as chemo-attractants while providing maturation signals to antigen-presenting cells such as dendritic cells (DCs) and macrophages [32–35]. Granulocytes can also attack helminth infections through antibody-dependent cell mediated cytotoxicity (ADCC), which implies the killing of antibody-coated parasites via the release of cytotoxic granules (degranulation). The degranulation is triggered by Fc-receptors (FcRs) recognizing antibody-bound antigen complexes on the cell surface and several cytokines mainly IL-3 and IL-5 [36,37]. Human granulocytes express FcγRI, FcγRIIa/b, FcγRIII, FcɛRI/II and FcαR and can be activated by IgGs, IgE and IgA. The activation of granulocytes can be measured *in vitro* by monitoring the expression of several activation markers including mainly CD63, a member of the tetraspan membrane glycoprotein family [38,39]. CD63 is an activation marker specific for neutrophils and basophils and, among other markers, for eosinophils [38,40,41] and it is responsible for the retention and sorting of pro-neutrophil elastase in the primary granules of neutrophils [38,40]. Upon degranulation, complete granule contents are released by fusion with the cellular membrane and cytolysis [42]. Granulocytes are characterized by six major granule proteins: major basic protein (MBP), eosinophil peroxidase (EPO), eosinophil cationic protein (ECP), eosinophil-derived neurotoxin (EDN), neutrophil elastase (NE) and histamine. MBP, EPO and ECP are potent helminth toxins [43,44]. These granule proteins have been shown to be involved in the killing of microfilariae of *Brugia spp*. and are associated in both allergy and helminth models with the development of immunopathology [45–49].

Since granulocytes play a central role in the elimination of large parasites, we hypothesized that antibodies present in plasma of EN, Mf+, Mf- and CP might differently impact granulocyte activation and functions. After comparing the suppressive properties of plasma and purified IgG antibodies from EN, Mf+, Mf- and CP, we found that granulocyte activation was significantly inhibited by plasma from EN and Mf+ individuals whereas plasma of Mf- and CP have no effect. This suppression was dependent on IgG4 in plasma of Mf+ whereas IgG-independent factors seem to be involved in EN. We have also demonstrated that IgG4 actively suppressed granulocyte activation and release of granule contents via FcγRI and FcγRII.

## Materials and methods

### Study population, samples collection and ethics

Patients and endemic controls’ samples were collected between 2008 and 2010 in villages of the Ahanta West and Nzema East Districts in the western region of Ghana endemic for LF (S1 Table). No other human filarial species were endemic in the region. The donors were recruited as part of a diagnostic and a clinical trial in LF (Clinical Trials Registration: ISRCTN15216778 and ISRCTN14757) [9,50,51]. Written informed consent was obtained from all participants. Persons eligible for participation were male adults in good health, 18–60 years of age, with a minimum body weight of more than 40 kg and without any clinical condition requiring chronic medication. Exclusion criteria included abnormal hepatic and renal enzyme levels (γ-glutamyltransferase > 28 U/L, glutamyl pyruvic transaminase > 30 U/L, creatinine > 1.2 mg/100 mL) assessed by dipstick chemistry, alcohol, drug abuse or antifilarial therapy in the past 10 months. Study participants were examined by a clinician using physical methods and a portable ultrasound machine (180 Plus; SonoSite, Bothell, WA) as described previously [51]. In addition, the presence of infections with other helminths (*Ascaris lumbricoides*, *Trichuris trichiura*, *Schistosoma spp*.) and protozoa (*Plasmodium*) was investigated using respectively Kato-Katz and finger prick tests. All samples included in the present study were free of such infections as previously described [9]. Ethical clearance was given by the Committee on Human Research Publication and Ethics at the University of Science and Technology in Kumasi, and the Ethics Committee at the University Hospital Bonn. Microfilarial load was determined by microscopic examination of fingerprick night blood samples as published [51]. Subsequently, 10 mL of venous blood was collected from each eligible volunteer and plasma was taken, aliquoted, and frozen at −80°C until used.

Samples included EN, residing in the endemic region but free of infection (CFA-, Mf-, n = 14), clinically asymptomatic microfilaraemic (CFA+, Mf+, n = 14) and amicrofilaraemic (CFA+, Mf-, n = 14) subjects, positive for circulating filarial antigen and a group of chronic pathological individuals with lymphedema and/or elephantiasis termed “CP” (n = 14), negative for filarial antigen. Also, plasma from European non-endemic blood donors (NEC, n = 14) was used as controls. Serum samples were obtained in an anonymized and de-identified form. All samples and controls used in the present study were randomly picked from the initial batches using a computer-based simple random algorithm as previously described [52]. Each sample in the initial batch was assigned a unique number and the samples corresponding to computer generated list were picked in each group and used in the study.

### Preparation of *Brugia malayi* antigen extracts

*Brugia malayi* worms recovered from the peritoneal cavity of jirds (*Meriones unguiculatus*) were obtained from NAID Filariasis Research Reagent Resource, FR3 (University of Georgia, Athens, GA). To prepare *B*. *malayi* antigen extract (BmAg), 100–300 frozen adult worms were thawed and transferred to a Petri dish pre-filled with sterile PBS (PAA, Pasching, Austria). Following several washes in PBS, worms were placed inside a glass mortar (VWR, Langenfeld, Germany). 3–5 ml of medium (RPMI without supplements) were added and worms were crushed until the solution was homogenous. The extract was then centrifuged for 10 minutes at 300 x g at 4°C to remove insoluble material. The supernatant was carefully transferred to a new tube. Protein concentration was measured using Bradford Assay. Aliquots were stored at -80°C until use. The extract was titrated to determine the optimal concentration for cell stimulation and the level of endotoxin was defined using the Pierce Limulus amoebocyte lysate (LAL) Chromogenic quantification kit (Thermo Fisher Scientific, Schwerte, Germany). The endotoxin level was below the detection limit of 0.1 EU/ml.

### Granulocyte purification from healthy blood donors

Granulocytes used in this study were purified from buffy coats of healthy European donors provided by the Institute for Experimental Haematology and Transfusion Medicine, University Clinic Bonn, Germany. Ethical clearance was given by the Ethics Committee of the University of Bonn (“Ethikkommission der Medizinischen Fakultät der Rheinischen Friedrich-Wilhelms-Universität Bonn”). Granulocytes were isolated using Ficoll-Hypaque (Pancoll, PAN Biotech, Aidenbach Germany) method. The density gradient was performed according to the manufacturer's instructions. Briefly, 15 mL heparinized venous blood samples were diluted with an equal volume of cold phosphate-buffered saline (PBS) in a 50 mL conical centrifuge tube, layered over Ficoll and centrifuged at 900 x g for 30 min at 4°C in a swinging bucket centrifuge (Thermo Scientific, Germany) with brake off. The opaque layer below the Ficoll/plasma interface containing granulocytes was transferred to another tube. After that, red cells were lysed by 10 min incubation at room temperature in 1x red blood cell lysis solution (Miltenyi Biotech, Bergisch Gladbach, Germany). Granulocytes were then centrifuged at 200 x g for 8 min at 4°C to remove contaminating red blood cells. Cell pellets were washed twice at 200 x g for 8 min in RPMI 1640 (Life Technologies, NY, USA). Supernatants were discarded and the purity of isolated granulocytes was assessed by flow cytometry. The purity was routinely ≥ 96%.

### Affinity chromatography for the purification of total IgG and IgG4

Total IgG was isolated from the plasma of EN, Mf+, Mf- and CP using prepacked HiTrap Protein G columns (GE Healthcare, Freiburg, Germany) according to the manufacturer’s instructions. Briefly, 100 μl of plasma samples were diluted with 1400 μl PBS and passed through a pre-equilibrated protein G-Sepharose column (GE Healthcare, Freiburg, Germany). Since Protein G binds to all human IgG subclasses, non-IgG plasma components were washed out from the column. Bound IgG was eluted in 1 ml fractions using IgG Elution Buffer (0.2 M Glycine/HCl, pH 3.0) and neutralized with saturated Tris-HCl (pH 9.0). The antibody concentration was then assessed at 280 nm using a NanoDrop 1000 spectrophotometer (Thermo Fischer Scientific, Wilmington, USA).

IgG4 antibodies were purified from IgG-enriched fractions using the CaptureSelect Human IgG4 affinity matrix (Life Technologies, Paisley, UK) according to the manufacturer’s instructions. Briefly, CaptureSelect affinity matrix was gently loaded and equilibrated in 10 ml affinity chromatography column with 1x PBS (pH 7.3). Diluted IgG-enriched fractions (1:1 volume PBS) were loaded onto the column and the linear flow rate was set at 15 cm/hour. After washing with 1x PBS, the column was eluted with 0.1 M Glycine (pH 3.0) and the fractions were immediately neutralized with Tris-HCl (pH 9.0). IgG4 fractions were collected and the purity of fractions assessed by determining the level of IgG subclasses, IgA, IgE and IgM antibodies by Luminex assay. In addition, the total protein concentration in plasma, IgG and IgG4 fractions was determined using a Bradford Protein Assay Kit (Thermo Fischer Scientific, Wilmington, USA).

### Affinity chromatography for the purification of human IgA

IgA purification was done using immobilized Peptide M/Agarose (InvivoGen, San Diego, USA) according to the manufacturer’s instructions. 1 ml of peptide M/Agarose gel was loaded into an appropriate microcentrifuge spin column (Thermo Scientific, Rockford, USA). 1 ml of IgG-depleted plasma from EN were then added to the column and incubated at room temperature for 30 min. After incubation, the column was washed and the flow-through was collected and labeled as IgA negative fractions (IgA-). The bound antibodies were then eluted with IgG elution buffer as described above. The resulting IgA positive (IgA+) fractions were neutralized immediately with neutralization buffer (1 M Tris–HCl, pH 9.0) and store at 4°C until use. Purity was assessed by Luminex analysis as described above. The purity was routinely > 90%

### Quantification of total protein in plasma and purified IgA, IgG and IgG4 fractions

To determine the protein concentration of plasma and purified IgA, IgG and IgG4 fractions, a Bradford protein assay kit (Thermo Scientific, Rockford, USA) was used according to the manufacturer’s instructions. In brief, serial dilutions of bovine serum albumin (BSA) was performed and used as standards against the samples. Another serial dilution of the samples was done in PBS. 300 μl per well of Coomassie blue G-250 (Cytoscelecton, Denver, USA) reagent was distributed in duplicate in a 96 well plate (Greiner Bio-One, Frickenhausen, Germany) and 3 μl of diluted samples and standard were added. The protein concentration in plasma, IgA, IgG and IgG4 fractions was then measured at 595 nm using a SpectraMAX 190 microplate reader (Molecular Devices, California, USA).

### Assessment of immunoglobulin isotypes expression in plasma and IgG/IgG4 positive and negative fractions

To analyze the isotype composition in IgA, IgG/IgG4 positive and negative fractions and in the plasma of EN and LF patients, ProcartaPlex Human Antibody Isotyping Panels (eBioscience, Vienna, Austria) were used according to manufacturer’s instructions. Briefly, antibody coated magnetic bead mixtures were incubated with 25 μl of assay buffer, kit standards or diluted plasma samples in a ProcartaPlex 96-wells plates at room temperature for 1 hour. 25 μl of detection antibodies mixture was then added and the plates were incubated on an orbital shaker at 500 rpm for 30 min. After that, each well was incubated with 50 μl of diluted Streptavidin-Phycoerythrin for 30 min. Plates were then washed using a hand-held magnetic plate washer. All incubations were performed at room temperature in the dark. Afterwards, samples were suspended in 120 μl reading buffer. Data were acquired using a MAGPIX Luminex system (Luminex Cooperation) and analyzed with ProcartaPlex Analyst software 1.0.

### Western blot analysis of eluted IgG fractions

The purity of eluted IgG fractions was analyzed by western blot. Samples were treated with 50 mM *2*-mercaptoethanol for 5 min, and equal quantities (2.5 μg) of the purified proteins and controls were loaded onto separate lanes of a polyacrylamide gel (10–12%) and resolved by SDS-PAGE (100 v, 45–60 min). The resolved proteins were transferred onto nitrocellulose membranes (GE Healthcare, Freiburg, Germany) using a Bio-Rad Trans-Blot Turbo Transfer system (Bio-Rad, Germany). The membranes were then blocked with gelatin blocking buffer (3% gelatin in Tris Buffered Saline (TBS)) (Bio-Rad, Germany) for 1 hour prior incubation with the primary antibody (polyclonal mouse anti-human IgG (H+L)) (Thermo Scientific, Rockford, USA) for 1.5 hours at room temperature. The nitrocellulose membranes were then washed with TBS/0.05% Tween 20 before incubation for 1 hour with alkaline phosphatase-conjugated goat anti-mouse IgG (Bio-Rad Laboratories, USA). Immune complexes were finally detected with NBT (nitro blue tetrazolium) and BCIP (5-bromo-4-chloro-3-indolyl-phosphate, Bio-Rad Laboratories, USA). Experiments were repeated at least three times.

### *In vitro* cell culture and granulocyte suppression assay

After establishing the optimal concentrations of IgE, anti-IgE and rIL-3 to induce granulocyte activation and degranulation (S1 Fig), the cells were purified as described above and 2 x 10^5^ cells/well were plated and pre-incubated with 40 ng/ml of natural human IgE antibody (Abcam, Cambridge, UK) for 30 min at 37°C/5% CO_2_ as previously described [53,54]. The cells were first preactivated at 37°C/5% CO_2_ for 10 min in the presence of 2 ng/ml rIL-3 (Miltenyi Biotech, Bergisch Gladbach, Germany), before being stimulated with 25 ng/ml anti-IgE mAb (Clone BE5) (Abnova, Taipei, Taiwan) and 10 μg/ml *Brugia malayi* Ag. Thereafter, the granulocytes were further incubated for 18 hours at 37°C/5% CO_2_ either alone (with culture medium), or in the presence of appropriately diluted plasma samples (5% v:v, containing 5 μg/ml of total proteins), IgG and corresponding IgG depleted fractions (5 μg/ml of total proteins) or 2.5 μg/ml IgG4 antibodies purified from the IgG positive fractions from the different clinical groups.

### Assessment of granule content release

Granulocyte culture supernatants were collected after 30 min and 18 hours to assess the level of histamine, and after 18 hours to investigate the release of ECP and NE using ELISA-Kits respectively from Abnova (Taipei, Taiwan), Abbexa (Cambridge, UK) and eBioscience (Vienna, Austria) according to the manufacturer’s recommendations. For histamine detection, samples and standards were first acylated by reacting 50 μl of samples, 25 μl of standards or control with 25 μl of acylation reagent and 25 μl of acylation buffer supplied in the test kit for 45 minutes. 25 μl aliquots of acylated standards, controls and samples were pipetted into wells of the antibody-coated microplate provided with the kit. Then the wells received 100 μl of histamine antiserum and the mixture was allowed to incubate for 3 hours at room temperature. The plates were then washed with the provided washing buffer to remove unbound materials. After that, the bound antibodies were detected using 100 μl of anti-rabbit IgG-peroxidase conjugate using TMB as a substrate. The color was allowed to develop for 20 minutes at room temperature in the dark. The reaction was stopped and the resulting OD values were measured at 450 nm. The histamine concentration, inversely proportional to the OD, was calculated using the SoftMax Pro Data Acquisition and Analysis Software.

For the assessment of ECP and NE, pre-coated ELISA plates were incubated with supernatants and standards for 1 hour at room temperature. The plates were then washed and incubated for 1 hour with HRP-conjugated anti-ECP and anti-NE polyclonal antibodies. After a final wash, the plates were developed using the provided TMB substrate and analyzed at 450 nm.

### Flow cytometry

To assess granulocyte activation and degranulation, the cells were harvested and washed with FACS buffer (PBS/2% FCS) at 1300 rpm for 8 min. 1x10^5^ cells were then resuspended in 100 μl of FACS buffer and blocked with 1 μl of FC- block (Affymetrix eBioscience, San Diego, CA, USA) for 15 min. 5μg/1x10^5^ cells of either anti-human CD66b-FITC (clone: G10F5) or CD63-PE (clone: H5C6) and HLADR-FITC (clone: LN3) (all from Affymetrix eBioscience) were then added and the cell suspension was incubated for 30 min at 4°C. Cells were then washed two times with FACS buffer and fixed in 200 μl PFA (4%). To correct spectral overlaps, fluorescence compensation was done using UltraComp ebeads (Affymetrix eBioscience). Data were acquired and analyzed using a FACS Canto flow cytometer and the BD-FACS-DIVA analysis software (BD Biosciences). Before the analysis, granulocyte viability was assessed by FACS using propidium iodide (PI-PE) and annexin-V (Annexin-FITC) (all from BD Biosciences, Heidelberg, Germany). All samples presented less than 1 and 10% necrotic cells respectively before and after 18 hours incubation.

### Cytospin and immunofluorescent staining for IgG4

Granulocytes were cultured as previously described and 2 x 10^5^ cells were harvested and washed with PBS. Then 100 μl of diluted cells were aliquoted into cytospin funnels and spun at 500 x g for 5 min onto glass slides (Engelbrecht, Edermünde, Germany) in a Hettich Cytospin centrifuge (Hettich, Tuttlingen, Germany) and immediately fixed in 4% PFA for 15 min. The slides were then blocked with PBS/1% BSA for 30 min and incubated with the primary antibody (mouse anti-human monoclonal IgG4) (Thermo Fischer Scientific, Rockford, USA) for 1 hour. After washing 3 times, the slides were incubated with the Alexa Fluor 488 coupled secondary antibody (goat anti-mouse polyclonal IgG antibody) (Thermo Fischer Scientific, Rockford, USA) for 1 hour at room temperature in a humidifying chamber. For the investigation of the Fc-receptors associated with IgG4-mediated granulocyte suppression, blocking antibodies against human FcγRI (2 μg/ml) (Biolegend, San Diego, CA, USA, clone:10.1), FcγRII (1 μg/ml) (Biolegend, San Diego, CA, USA, clone: FUN-2) and FcγRIII (4 μg/ml) (Biolegend, San Diego, CA, USA, clone: 3G8) were added before the incubation with purified IgG4 antibodies. Nuclear DNA was labeled with 0.25 μg/ml DAPI (Thermo Fischer Scientific, Rockford, USA) in PBS for 5 min. Cells were then mounted in VECTASHIELD-Antifade mounting medium (Vector Laboratories, CA, USA) and the slides were analyzed using a Zeiss LM-Set Axiocam MRm microscope (Carl Zeiss, Thornwood, NY, USA).

### Antigen-specific ELISA

To evaluate the capacity of purified IgG4 from EN, Mf+, Mf- and CP individuals to interact with Brugia antigen, 50 μl Brugia antigen (10 μg/ml) were coated on high binding ELISA plates (Greiner Bio-One, Frickenhausen, Germany) overnight at 4°C. The plates were then washed 5 times with PBS/0.05% Tween 20 and blocked with PBS/1% BSA for 1 hour at room temperature. The wash step was repeated and 50 μl/well of purified IgG4 from EN, Mf+, Mf- and CP (2.5 μg/ml) were added and the plates were incubated again at 4°C overnight. The wells were washed again as described above and diluted biotin-conjugated mouse anti-human IgG4 (clone JDC-14) (1:1000) (from BD Biosciences, Heidelberg, Germany) was added, followed by incubation at room temperature for 2 hours. After an additional washing step, the plates were incubated with 50 μl/well of Streptavidin-HRP for 45 min in the dark. After a final washing step, 50 μl/well TMB substrate solution were added and the reaction was stopped with 25 μl/well 2N H2SO4 (Merck KGAA, Darmstadt, Germany). Optical density was measured at 450 nm using the SpectraMAX ELISA reader and the results were expressed as arbitrary units (AU) using as a standard a plasma sample arbitrarily set at 5 AU.

### Statistical analysis

All statistical analyzes were performed using Prism 5.03 software (GraphPad Software, Inc., La Jolla, USA). Comparative analyzes among groups were conducted using the Kruskal-Wallis test with a Dunn’s nonparametric post-hoc test (> 2 groups). Significance was accepted when p < 0.05. Correlation between the levels of antibodies in IgG negative fractions and inhibition capacity was analyzed using Spearman’s rank correlation.

## Results

### IgG4 and IgA are characteristic for Mf+ and EN respectively, whereas IgE is prominent in Mf- and CP

**Fig 1:**
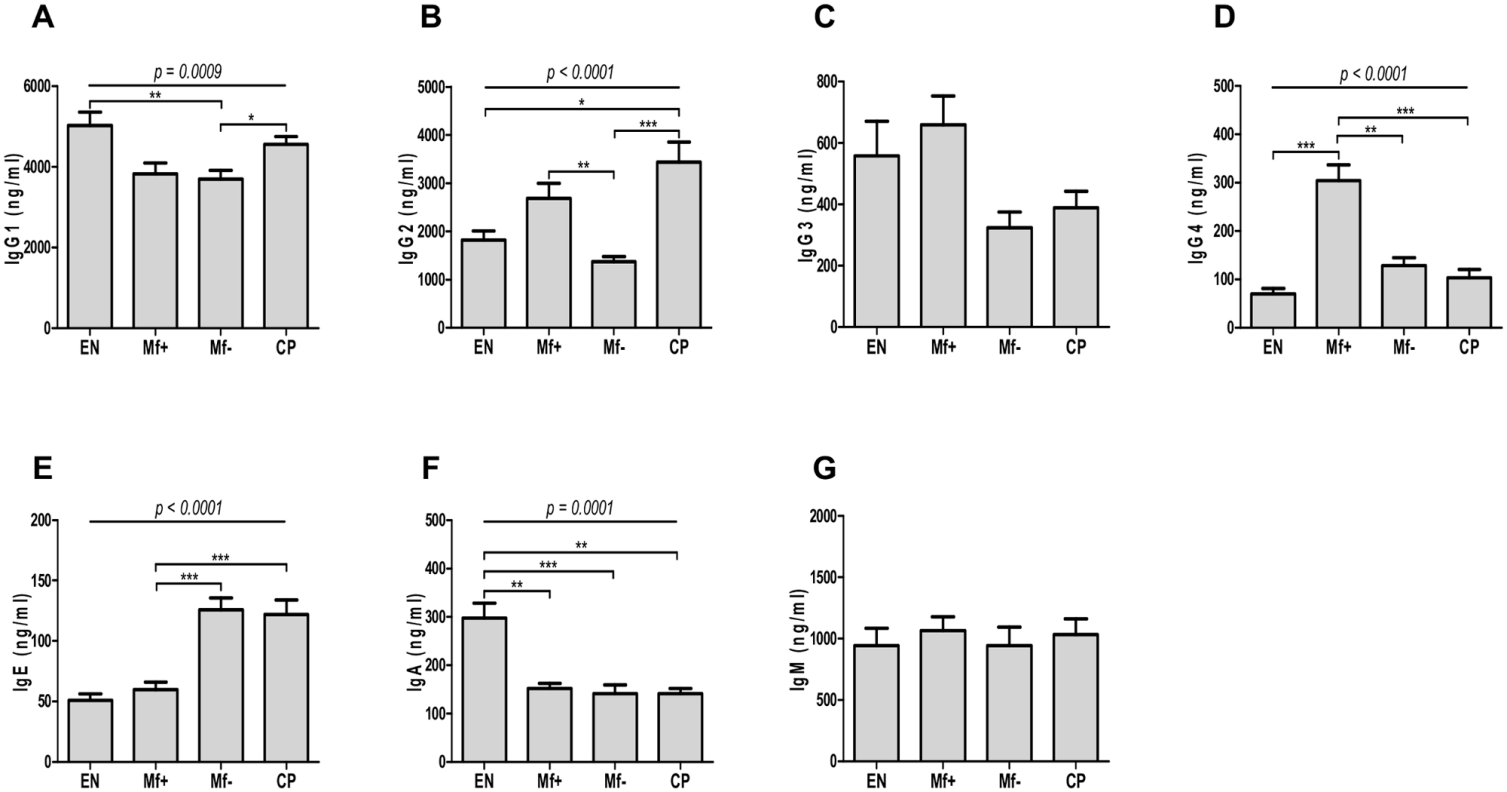
Preferential expression of IgG4 in Mf+. Plasma samples from EN (n = 14) and LF infected Mf+ (n = 14), Mf-(n = 14) and CP (n = 14) patients were diluted and analyzed for the expression of IgG1 (A), IgG2 (B), IgG3 (C), IgG4 (D), IgE (E), IgA (F) and IgM (G) using Luminex-based immunoassay. Bars depict the plasmatic antibody expressions as mean ± SEM. Statistical comparison was based on Kruskal-Wallis one-way ANOVA followed by Dunn post-hoc test. The indicated p-values refer to the significance level among all groups and asterisks indicate the level of significance, determined by Dunn’s multiple comparisons test; *: p<0.05; **: p<0.01; ***: p<0.001.

To define the initial antibody profile of EN, Mf+, Mf- and CP, we compared the plasma levels of IgG1, IgG2, IgG3, IgG4, IgE, IgM and IgA in different groups using a Luminex-based immunoassay. We found that the IgG1 expressions observed in EN and CP were similarly high. In contrast, Mf+ and Mf- individuals presented reduced IgG1 levels (Fig 1A). However, while the highest levels of IgG2 were detected in the plasma of CP individuals, plasmatic IgG2 in EN and Mf- were significantly lower compared to Mf+ and CP (Fig 1B). The differences observed in the expression of IgG3 between the four groups were not statistically significant (Fig 1C). Interestingly, the expression of IgG4 was relatively low in EN, Mf- and CP but significantly elevated in Mf+ (Fig 1D). This contrasts with lower levels of IgE in those patients in comparison to Mf- and patients with chronic pathological manifestations (Fig 1E). In addition, plasma of EN expressed higher IgA levels compared to Mf+, Mf- and CP (Fig 1F), whereas no significant differences were seen in the expression of IgM (Fig 1G).

### Plasma from EN and Mf+ but not those of Mf- and CP inhibits granulocyte activation and degranulation

**Fig 2:**
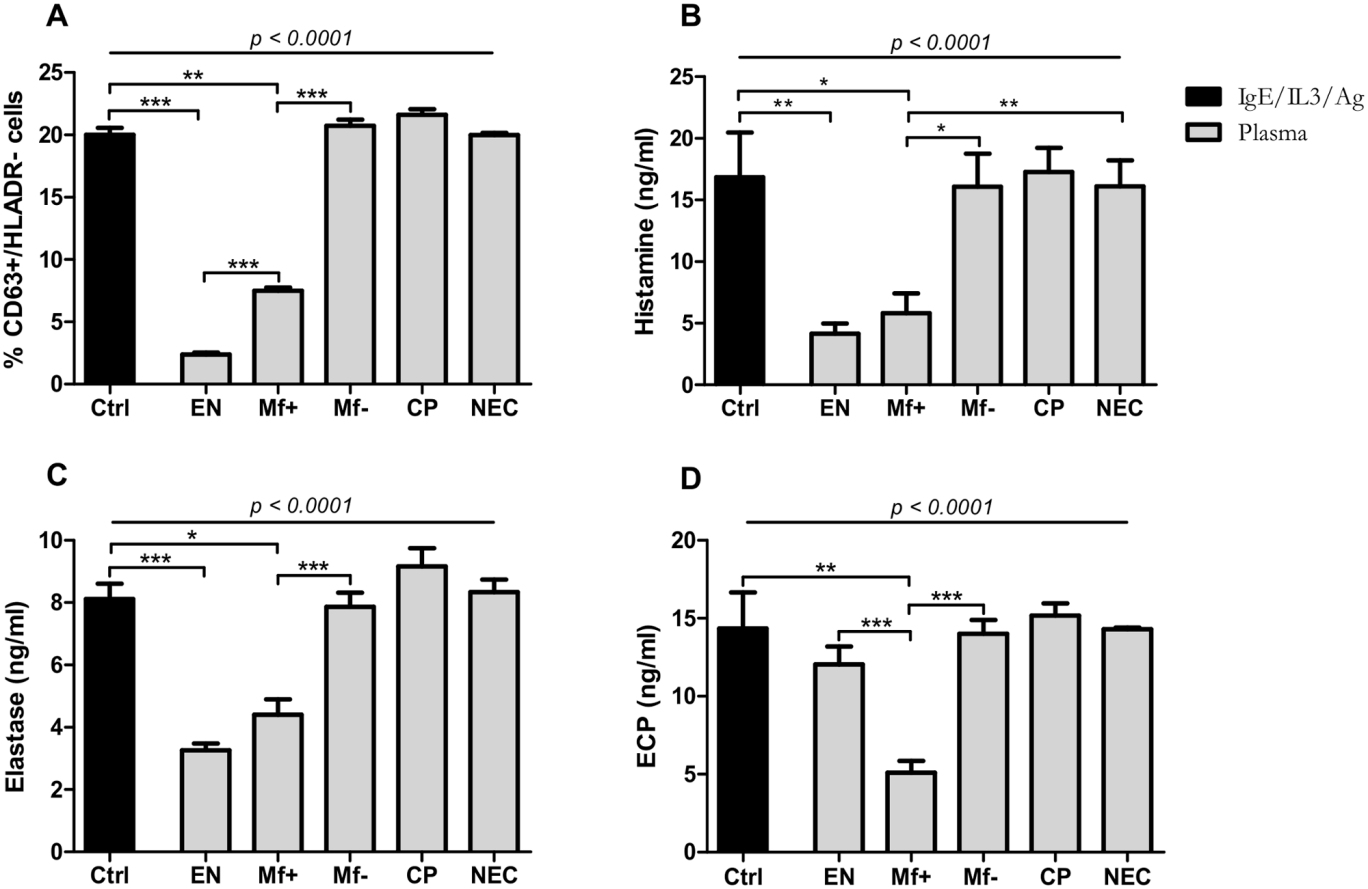
Plasma from EN and Mf+ patients suppressed granulocyte activation and degranulation. Freshly isolated granulocytes from healthy blood donors (n = 9) were stimulated with IL-3 (2 ng/ml), anti-IgE (25 ng/ml) and Brugia antigen extracts (10 μg/ml) as control (dark bars) and then cultured in presence of 5% (v/v) of plasma (with a final concentration of 5 μg/ml of total proteins) of either non-endemic controls (NEC), endemic normal (EN), microfilaria positive (Mf+), microfilaria negative individuals (Mf-) or plasma of patients with chronic pathology manifestations (CP) (grey bars). Cells were then stained for CD63 and HLADR antigens expression. The proportion of activated granulocyte cells (CD63+/HLADR- cells) was determined after 18 hours of incubation (A). The release of histamine after 30 min (B), neutrophil elastase (C) and eosinophil cationic protein (D) after 18 hours was measured in culture supernatants. Bars represent means ± SEM. Graphs are representative of 3 independent experiments. Statistical comparison was based on Kruskal-Wallis one-way ANOVA followed by Dunn post-hoc test. The indicated p-values refer to the significance level among all groups according to Kruskal-Wallis test. Asterisks indicate the level of differences after Dunn’s multiple comparisons test; *: p<0.05; **: p<0.01; ***: p<0.001.

Because plasma samples from Mf+ individuals presented high levels of IgG4 antibody and since IgG4 antibodies are known to exhibit anti-inflammatory properties, we hypothesized that plasma from Mf carriers, and specifically IgG4 molecules, would preferentially down-modulate granulocyte activation and degranulation. We then next investigated how crude plasma of NEC, EN, Mf+, Mf- or CP modulates the function of IL-3/anti-IgE/BmAg activated granulocytes by monitoring the expression levels of CD63/HLADR and analyzing the release of granule components (histamine, ECP and NE). While plasma from NEC, CP and Mf- had no effect on granulocytes (Fig 2 and S2 Fig), those from EN and Mf+ significantly inhibited activation of granulocytes as indicated by the lower percentages of CD63+HLADR- cells (Fig 2A). Interestingly, the plasma of EN presented a higher inhibitory potential on granulocyte activation when compared to those of Mf+. In line with the activation data, plasma from both EN and Mf+ significantly suppressed the release of histamine (Fig 2B and S2 Fig) and NE (Fig 2C). In addition, histamine levels were higher after 30 min and were lower, but detectable, after 18 hours. However, while the plasma of Mf+ individuals significantly inhibited the release of ECP in granulocyte cultures, those of EN failed to suppress the release of ECP (Fig 2D). These results indicate that, in lymphatic filariasis, active factors in EN and Mf+ infected patients’ plasma environment but not present in Mf- and CP patients impaired granulocyte activation.

### Depletion of IgG reduces the suppressive capacity of plasma from Mf+ but not of EN

**Fig 3:**
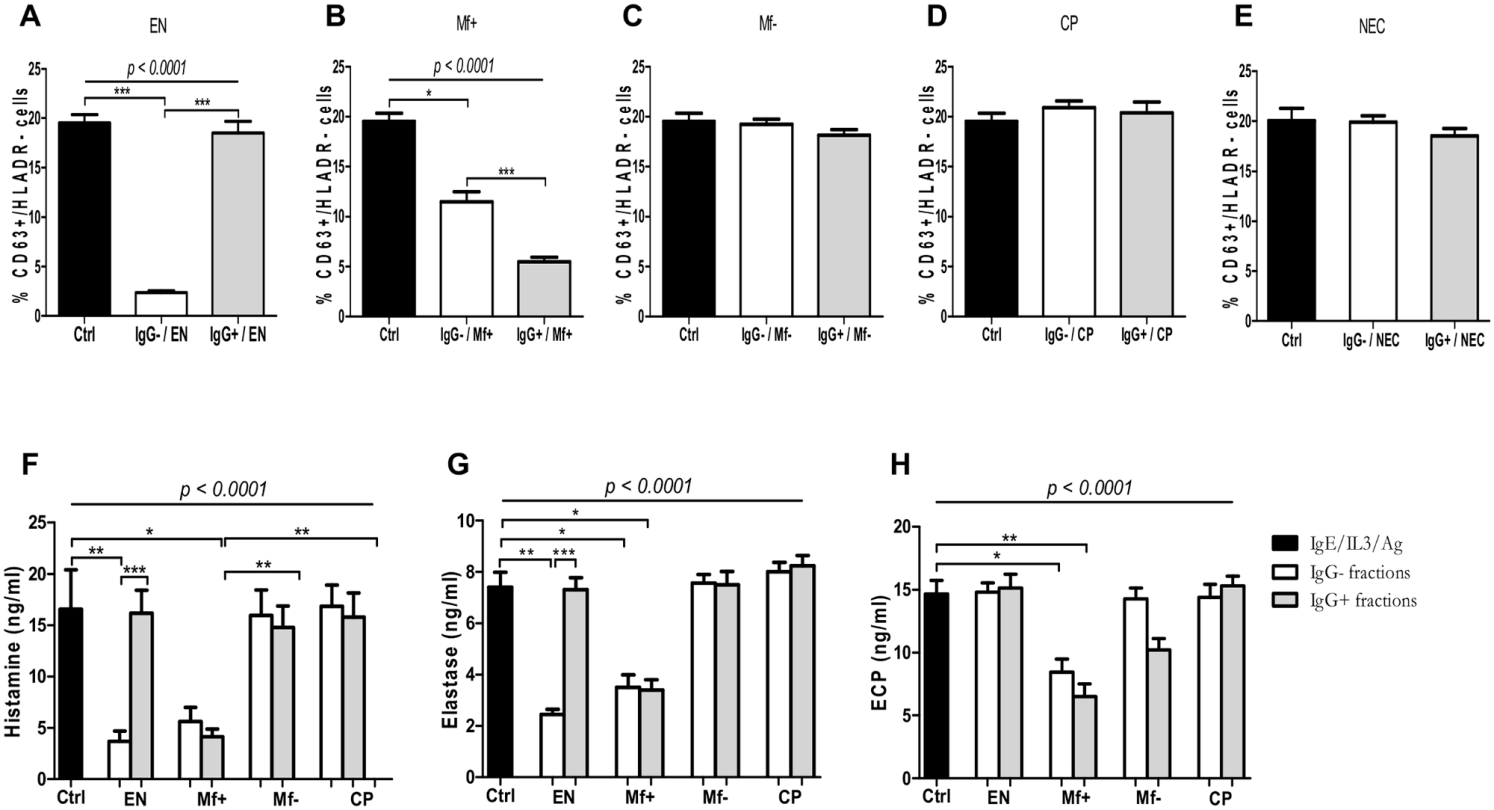
Suppression of granulocyte activation and degranulation is IgG-independent in EN but IgG-dependent in Mf+. Freshly isolated granulocytes from healthy blood donors (n = 9) were stimulated with IL-3 (2 ng/ml), anti-IgE (25 ng/ml) and Brugia antigen extracts (10 μg/ml) alone (dark bars) or in presence of 5 μg/ml of IgG negative fractions (n = 8) (light bars) from EN (A), Mf+ (B), Mf- (C), CP (D) and NEC (E) or the corresponding IgG positive fractions (grey bars). After that, cells were stained for CD63 and HLADR expression. Activated granulocytes were characterized as CD63+/HLADR- cells. Bars represent means ± SEM of the percentage of activated granulocyte CD63^+^/HLADR^-^ cells. The release of histamine after 30 min (F), neutrophil elastase (G) and eosinophil cationic protein (H) after 18 hours in culture supernatants was assessed. Graphs are representative of 3 independent experiments. Statistical comparison was based on Kruskal-Wallis one-way ANOVA followed by Dunn post-hoc test. The indicated p-values refer to the significance level among all groups and asterisks indicate the level of significance, determined by Dunn’s multiple comparisons test; *: p<0.05; **: p<0.01; ***: p<0.001.

To define the role of IgGs in the suppression of granulocytes by plasma of EN and Mf, we depleted IgG antibodies per affinity chromatography. The purity was analyzed (S3 Fig), and the ability of IgG positive and negative fractions to modulate granulocyte activation was tested. Interestingly, while IgG negative (IgG-) fractions of EN significantly suppressed granulocyte activation, IgG positive (IgG+) fractions showed no effect (Fig 3A). Both IgG+ and IgG- fractions from Mf+ significantly inhibited granulocyte activation (Fig 3B) whereas neither IgG+ nor IgG- fractions from NEC, Mf- and CP affected granulocyte activation (Fig 3C–3E). Moreover, in Mf+, the IgG-related inhibition was significantly higher than that observed with negative fractions. These trends were also reflected in the release of histamine (Fig 3F) and NE (Fig 3G). Surprisingly both fractions from EN did not impair ECP release (Fig 3H) when compared with histamine and NE. These data suggest that whereas total IgG from Mf+ individuals inhibited granulocyte activation, IgG-independent factors, seem to be involved in the suppression by plasma of both EN and Mf+ individuals.

### IgA expression is associated with the suppressive capacity of IgG-negative fractions from EN

**Fig 4:**
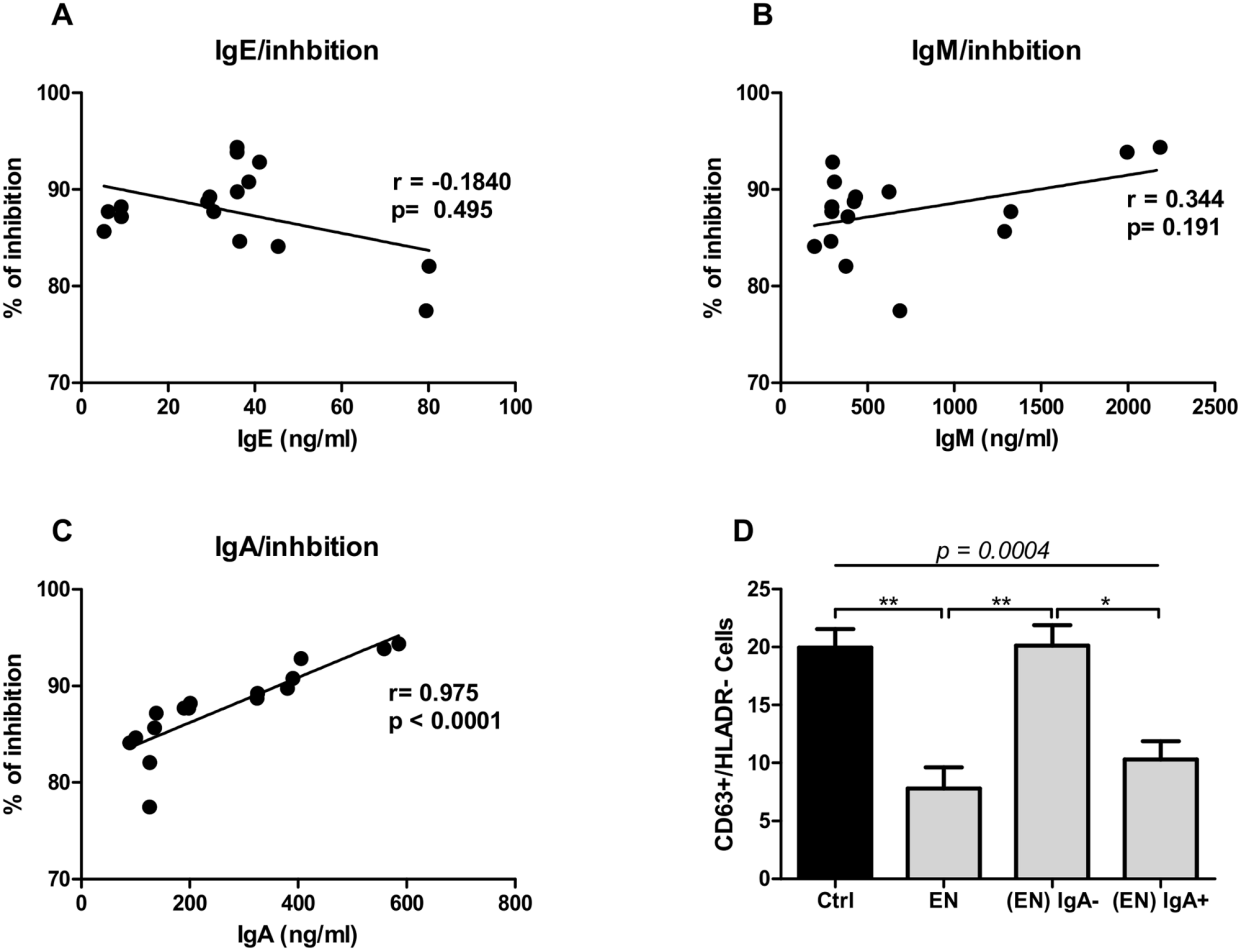
IgA expression correlated with the suppressive capacity in the IgG-negative fractions of EN. Freshly isolated granulocytes from healthy blood donors (n = 9) were stimulated with IL-3 (2 ng/ml), anti-IgE (25 ng/ml) and Brugia antigen extracts (10 μg/ml) alone or in the presence of 5 μg/ml of IgG negative fractions of EN. Cells were stained with anti-CD63 and HLADR antibodies. Activated granulocytes were characterized as CD63+/HLADR- cells and the percentage of inhibition was calculated using the control without plasma as 100% of activation (0% of inhibition). The percentage of inhibition was then correlated with the expression of IgE, IgM and IgA determined by Luminex assay. Each dot represents the plot of the expression of IgE (A), IgM (B) or IgA (C) to the corresponding value of the inhibition. Thereafter, the percentage of activated granulocytes (CD63+/HLADR- cells) was determined after 18 hours of incubation (D) in the presence of IgE/IL-3 alone (dark bar) or in combination with either bulk plasma from EN (EN), IgA negative fractions (EN) IgA- or IgA positive fractions (EN) IgA+. Bars represent means ± SEM of the percentage of activated granulocyte CD63^+^/HLADR^-^ cells. Statistical comparison was based on Spearman’s rank correlation (A-C) or Kruskal-Wallis one-way ANOVA followed by Dunn post-hoc test (D). The indicated p-values refer to the significance level among all groups and asterisks indicate the level of significance, determined by Dunn’s multiple comparisons test; *: p<0.05; **: p<0.01. Graphs are representative of 3 independent experiments.

To define the IgG-independent factors responsible for granulocyte suppression in EN, we correlated the expression of the remaining antibodies in the IgG-negative fractions (IgA, IgE and IgM) with the ability of these plasma to inhibit granulocyte functions. Interestingly, while IgE and IgM presented no correlation with the inhibitory capacity of IgG-negative fractions of EN (Fig 4A and 4B), IgA expression significantly correlated with the inhibition capacity on activated granulocytes (Fig 4C). In addition, while IgA-depleted fractions [(EN) IgA-] lose their ability to suppress granulocyte activation as shown by similar expression of CD63+ cells when compared to the control, peptide M purified IgA+ fractions significantly reduced granulocyte activation (Fig 4D). These data strongly suggest that IgA expression in EN is associated with the inhibition effect observed when their plasma were incubated with activated granulocytes.

### IgG4 antibodies purified from EN, Mf+, Mf- but not CP dampen granulocyte functions in a dose-dependent manner

**Fig 5:**
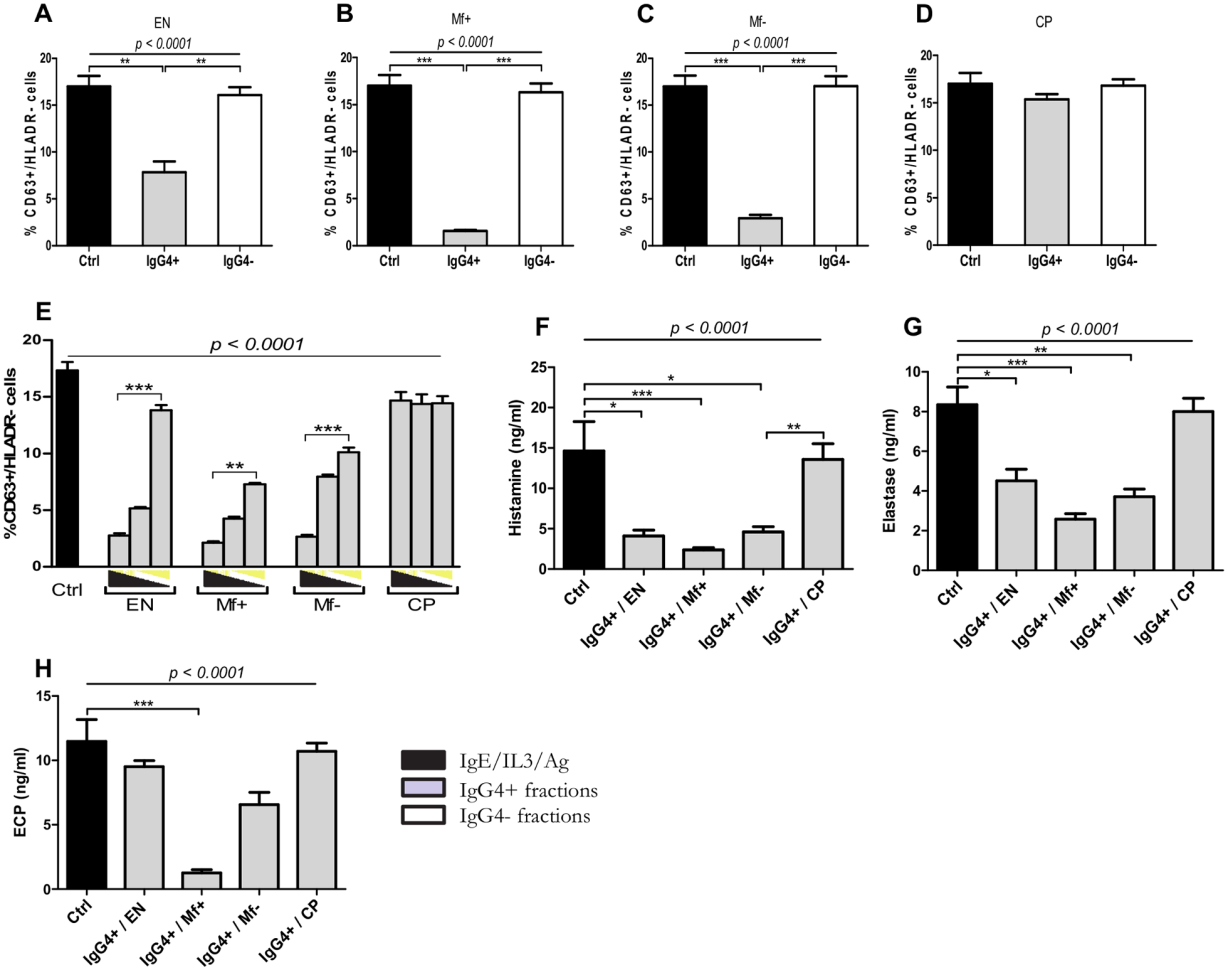
Depletion of IgG4 abrogates the suppressive capacity of IgG positive fractions from plasma of LF infected individuals. Freshly isolated granulocytes from healthy blood donors (n = 9) were stimulated with IL-3 (2 ng/ml), anti-IgE (25 ng/ml) and Brugia antigen extracts (10 μg/ml) alone (dark bars) or in presence of 2.5 μg/ml of IgG4 negative fractions obtained from IgG enriched fractions (n = 8) (light bars) from EN (A), Mf+ (B), Mf- (C), CP (D) or the corresponding IgG4 positive fractions (n = 8) (grey bars) and increasing concentration (1.25 μg/ml, 2.5 μg/ml and 5 μg/ml) of IgG4 positive fractions from different groups (E). Cells were stained with anti-CD63 and HLADR antibodies. Activated granulocytes were characterized as CD63+/HLADR- cells. The release of histamine (F), neutrophil elastase (G) and eosinophil cationic protein (H) in supernatants from cultures with IgG4 positive fractions was assessed after 18 hours. Bars represent means ± SEM. Graphs are representative of 3 independent experiments. Statistical comparison was based on Kruskal-Wallis one-way ANOVA followed by Dunn post-hoc test. The indicated p-values refer to the significance level among all groups according to Kruskal-Wallis test. Asterisks indicate the level of differences after Dunn’s multiple comparisons test;*: p<0.05; **: p<0.01; ***: p<0.001.

We next investigated whether the modulation of granulocyte activation and degranulation by Mf+ IgG fractions is associated with the presence of the anti-inflammatory isotype IgG4. Highly pure fractions of IgG4 antibodies were prepared and the purity validated (S4 Fig). Thereafter the purified fractions were tested on activated granulocytes. Strikingly, while IgG4 antibodies from EN, Mf+ and Mf- significantly suppressed granulocyte activation (Fig 5A–5C and S4 Fig), those from CP have failed to suppress granulocyte activation (Fig 5D) compared to the control. In addition, the suppressive effect was completely abrogated after IgG4 removal from IgG fractions (Fig 5A–5C), suggesting that the suppressive effect of IgG fractions stems from IgG4 molecules and not from other IgG antibodies. Furthermore, we investigated whether these effects were dose-dependent. Whereas increasing concentrations of IgG4 from EN, Mf+ and Mf- proportionally reduced the percentage of activated cells (CD63+/HLADR-) in a dose-dependent manner, no dose effect was seen when IgG4 from CP patients were used (Fig 5E). Interestingly, the suppressive effect of IgG4 affected mostly neutrophils and basophils but not eosinophils (S5 Fig). Consistent with the granulocyte activation data, we detected lower levels of histamine and NE in supernatants of granulocyte cultures treated with IgG4 antibodies from EN, Mf+ and Mf- compared to those treated with CP-IgG4 (Fig 5F and 5G). However, no significant reduction in the release of ECP was observed after incubation with IgG4 from EN or Mf- (Fig 5H). Table 1 summarizes the effects of plasma, IgG and IgG4 fractions on granulocyte activation.

**Table 1:** Effect of plasma and purified IgG and IgG4 fractions from EN, Mf+, Mf- and CP on granulocyte activation.

			Fractions		
	Crude plasma	Total IgG	IgG neg	IgG4 pos	IgG4 neg
**EN**	**S**	**NS**	**S**	**S**	**NS**
**Mf+**	**S**	**S**	**S**	**S**	**NS**
**Mf-**	**NS**	**NS**	**NS**	**S**	**NS**
**CP**	**NS**	**NS**	**NS**	**NS**	**NS**

Plasma, IgG and IgG4 fractions from EN, Mf+, Mf- and CP individuals were incubated with granulocytes and the effect on granulocyte activation was investigated after 18 hours culture. S = Suppression of granulocytes; NS = No suppression of granulocyte functions; EN = Endemic normal; Mf+ = Microfilaria positive; Mf- = Microfilaria negative; CP = Chronic pathology

### IgG4 antibodies modulate granulocyte activities via FcγRI and II

**Fig 6:**
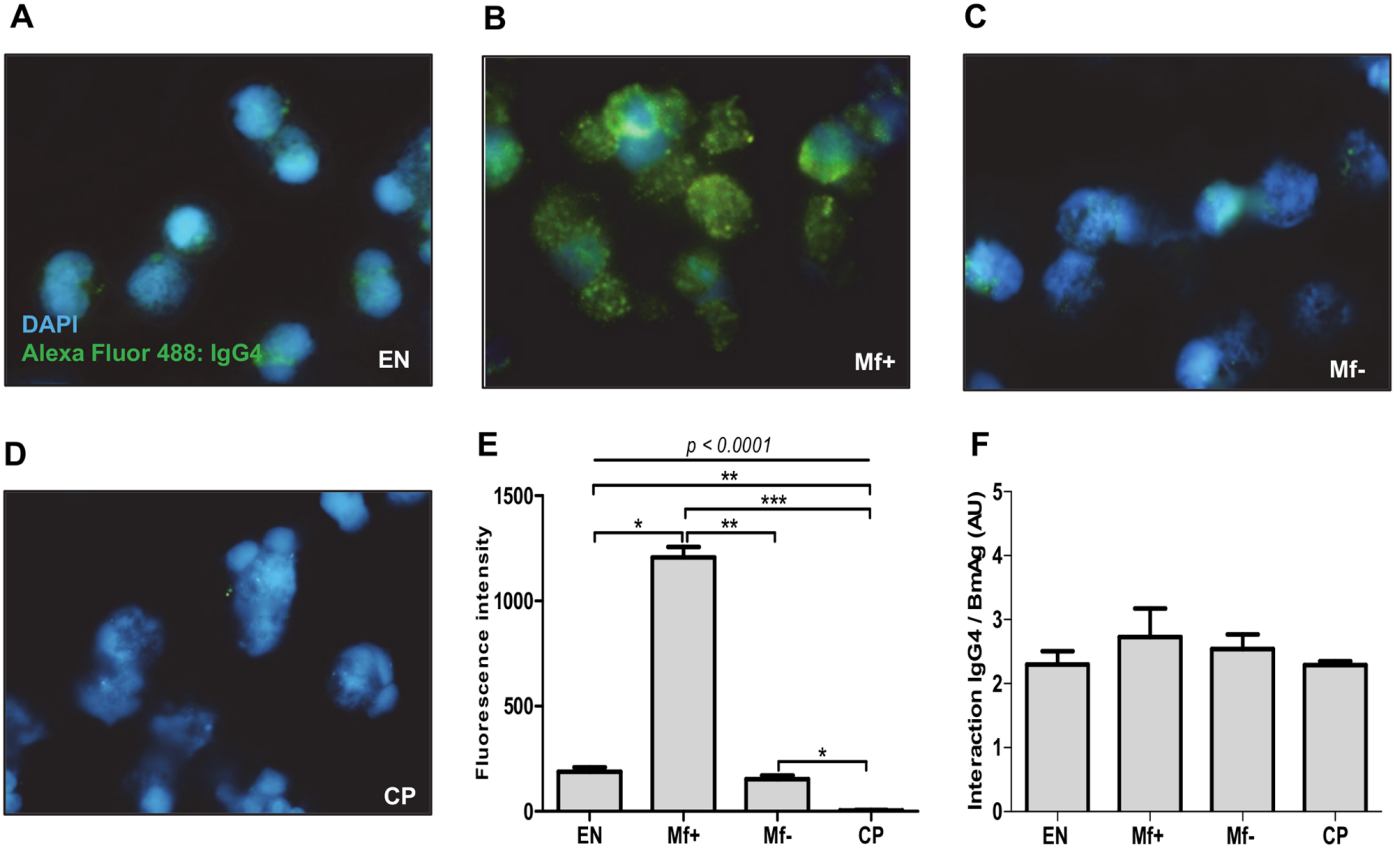
IgG4 antibodies from Mf+ patients present a higher affinity for granulocytes compared to IgG4 from EN and Mf-. Purified granulocytes from healthy blood donors were stimulated with IL-3 (2 ng/ml), anti-IgE (25 ng/ml), and Brugia antigen extracts (10 μg/ml) and cultured in presence of 2.5 μg/ml of IgG4 molecules from EN (A), Mf+ (B), Mf- (C) and CP (D) for 18 hours. The cells were then stained with DAPI (blue) and IgG4 binding on activated cells was revealed with Alexa fluor 488 labelled antibody (green). Original magnification x100. The median green fluorescence intensities are illustrated in E. A representative experiment of 3 is shown. Thereafter, the relative affinity of IgG4 from EN, Mf+, Mf- and CP with Brugia antigen was analyzed by ELISA (F). Statistical comparison was based on Kruskal-Wallis one-way ANOVA followed by Dunn post-hoc test. The indicated p-value refers to the significance level among all groups according to Kruskal-Wallis test. Asterisks indicate the level of differences after Dunn’s multiple comparisons test; *: p<0.05; **: p<0.01; ***: p<0.001.

To further explore the mechanisms by which IgG4 interfere with granulocyte activities, we examined the ability of purified IgG4 antibodies from each group to bind to granulocytes and Brugia antigen. While IgG4 molecules from EN, Mf+ and Mf- were able to interact with effector cells, those from CP had no affinity to granulocytes (Fig 6). However, IgG4 from Mf+ presented a much higher affinity for the cells in comparison to those from Mf- and EN (Fig 6A–6C and 6E). Since differences in the affinity of IgG4 from EN, Mf+, Mf- and CP to form complex with Brugia antigen can also affect granulocyte modulation, we analyzed the capacity of IgG4 from the different groups to interact with Brugia antigen. We detected no significant differences in the capacity of IgG4 from EN, Mf+, Mf- and CP to form a complex with Brugia antigen (Fig 6F).

**Fig 7:**
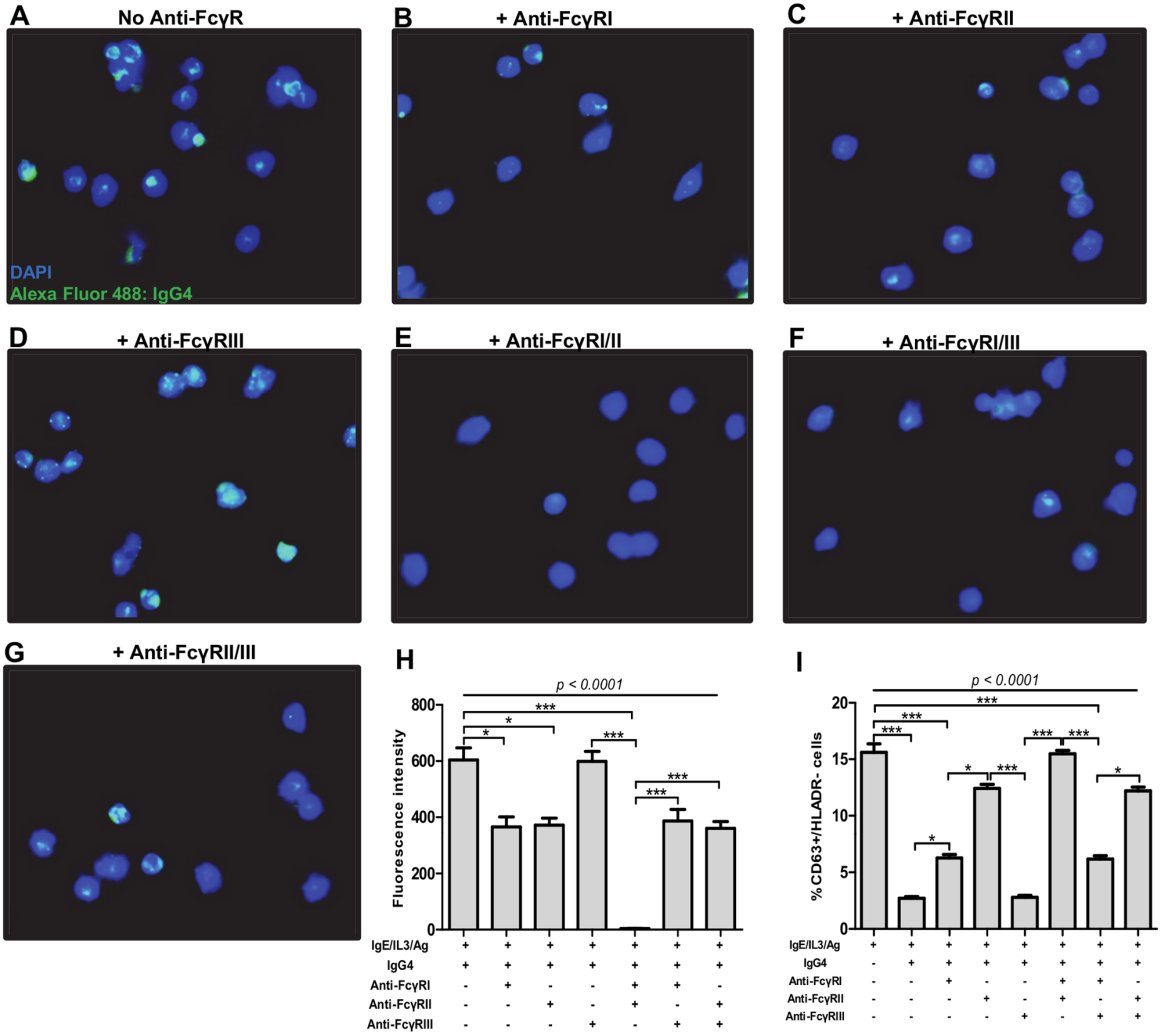
Anti-inflammatory IgG4 antibodies modulate granulocyte functions via FcγRI and FcγRII. Granulocytes from healthy blood donors were purified and stimulated with IL-3 (2 ng/ml), anti-IgE (25 ng/ml), and Brugia antigen extracts (10 μg/ml) and incubated with either medium or anti-FcγRI (2μg/ml), FcγRII (1 μg/ml) or FcγRIII (4 μg/ml) antibodies alone or in combinations. Thereafter granulocytes were incubated with 2.5 μg/ml of affinity purified IgG4 for 18 hours. The cells were then stained with DAPI (blue) and the presence of IgG4 was detected with anti-IgG Alexa fluor 488 antibody (green). A representative experiment out of 6 is shown: A-G are representative fluorescence microscopy images of granulocytes incubated with culture medium (A) or in the presence of blocking antibodies against FcγRI (B), FcγRII (C), FcγRIII (D) or a combination of antibodies against FcγRI and II (E), FcγRI and III (F), or FcγRII and III (G). Bars represent mean fluorescence intensities ± SEM (H) or the percentages of CD63+/HLADR- activated granulocytes (I) in 6 independent experiments. Statistical comparison was based on Kruskal-Wallis one-way ANOVA followed by Dunn post-hoc test. The indicated p-values refer to the significance level among all groups according to Kruskal-Wallis test. Asterisks indicate the level of differences after Dunn’s multiple comparisons test; *: p<0.05; **: p<0.01; ***: p<0.001.

Because IgG4 from Mf+, Mf- and EN bound to granulocytes, we next investigated which FcγRs are involved in their fixation by using blocking antibodies against FCγRI, FCγRII, and FCγRIII. We observed that the blockade of FcγRI (Fig 7B and 7H) and FcγRII (Fig 7C and 7H) but not FcγRIII (Fig 7D and 7H) significantly reduced IgG4 binding to granulocytes. Interestingly, the capacity of IgG4 to bind to granulocytes was completely abrogated when FcγRI and FcγRII were simultaneously blocked (Fig 7E and 7H). Corresponding results were also observed when the activation of granulocytes in the presence of IgG4 and anti-FCγRs was measured (Fig 7I). These findings suggest that IgG4-mediated granulocyte suppression in Mf+ patients involves FcγRI and FcγRII but not FcγRIII.

## Discussion

The pathology of lymphatic filariasis results from the complex interplay between the pathogenic potential of the parasite, the host’s immune response and collateral bacterial and/or fungal infections [55]. Even though the role of IgG4 in immunosuppression in filariasis is well known [56], only few data exist on their impact on effector cells. Recent investigations suggest that different granulocyte subsets may be important in the immune response to helminth infections [28,29]. Here we used a sequential depletion/purification approach to define the immune components that are responsible for granulocyte suppression in the plasma of individuals from the different LF clinical groups. The advantage of this approach is that, in addition to analysing quantitative differences among these groups, it can be investigated whether a given antibody retains functional capacity when used at normal concentrations. We tested both IgG positive and negative fractions and could show that while IgG+ fractions from Mf+ suppressed granulocytes, those of EN, Mf- and CP had no effect. A further purification step on the IgG+ fractions using anti-IgG4 in the purification matrix indicated that IgG4+ fractions from Mf+, Mf- and EN displayed comparable suppressive capacities. This apparent contradiction with the data obtained using plasma and total IgG is due to the level of purification of IgG4 used here at the same concentration and confirms that quantitative differences in the expression of IgG4 play a significant role in the suppressive properties observed in the plasma of the different clinical groups. These data suggest that IgG4 antibodies from EN, Mf+ and Mf- might have the same overall suppressive properties and the alterations observed when using total IgG or crude plasma are due to differences in the ratios IgG4/total IgG as previously postulated [10,57]. These observations are in line with findings of Mohapatra et al., indicating that plasma of asymptomatic individuals (Mf+) in contrast to those of CP mediated suppression of mitogen-induced proliferation of human PBMCs [58]. Bennuru et al. further demonstrated that sera from CP patients promoted the proliferation of lymphatic endothelial cells whereas those of EN suppressed this proliferation [59]. Plasma of Mf- and CP contain higher levels of pro-inflammatory IgG1-3 and IgE antibodies, known to be relevant for parasite clearance as demonstrated in different animal models of filariasis [60–66] but are also associated with pathology development in CP-patients [4,9,67].

The data obtained using the plasma of CP patients were initially similar to those for EN and Mf-, since they displayed low levels of IgG4 and presented no inhibition effect on granulocytes. However purified IgG4 from this clinical group fundamentally lack suppressive properties even when higher concentrations were used, suggesting the existence of two distinct mechanisms of inhibition of the immunoregulatory antibody IgG4 in CP patients, first by down-modulating its level and second by modifying the properties of the remaining IgG4 molecules. This is likely associated with the proinflammatory environment including Th17 and Th22 characteristic of the CP profile [68]. A recent study on patients undergoing cardiac surgery indicated a strong connection between glycosylation features related to fucosylation, sialylation and bisecting GlcNAc and severity of inflammatory response [69]. Since glycosylation features can affect the function of antibodies [70], this is a possible explanation for the lack of suppressive properties of IgG4 antibodies purified from CP patients. Since no differences were detectable in the purity of the IgG4 positive fractions, and because IgG4 is known to present no allotypic variations [71], post-translational alterations could, as mentioned above, explain why functional differences are observed between IgG4 molecules in our settings. Indeed, different investigations have shown that all IgGs contain a conserved glycosylation site at N297 in CH2 domain that is important for the structural conformation of the Fc region necessary for binding to FcRs and complement factors [71–73]. Differences in the glycosylation states may ultimately influence the effector pathways elicited by the Fc domain [73]. Fucosylation and sialylation for example are two extensively investigated glycan modifications of Fc that significantly modulate the affinity of Fc regions to FcRs [70,73]. In several health and disease settings, a shift toward certain Fab- and Fc-glycoforms of antibodies has been reported [70]. It is very likely that the degree of glycosylation differs in the IgG4 molecules from EN, Mf+, Mf- and CP and subsequently modulates their affinity to FcγRs.

In addition to post-translational alterations, other factors such as the previously described differential recognition of filarial antigens by antibodies in the different clinical groups [74], could also have an impact on the ability of IgG4 antibodies to bind granulocytes. Indeed, differential recognition of filarial antigens could differently affect the formation of antigen-antibody complexes and thus modulate the inhibition capacity of IgG4 on activated granulocytes. Other parameters that potentially can explain the different affinity and inhibition capacity of IgG4 antibodies from the four LF clinical groups are FcR cross-linking and steric hindrance that can respectively reduce availability and access to FcγRs on granulocyte surface [75]. Differences in the inhibition effect could also be modulated by differences in the levels of autoantibodies in the different clinical groups as suggested by Mishra et al. [76].

The most unexpected finding in the present work is the suppression of granulocyte activation by the plasma of EN since this clinical group is usually associated with putative immunity and strong pro-inflammatory responses [77]. Our data indicated an elevated expression of IgA in the plasma of EN and revealed a significant correlation between IgA expression and the suppressive properties in the IgG-negative fractions of EN. Sahu et al. observed similar trends when comparing the expression of filarial-specific IgA in LF endemic populations [78]. In addition, recent investigations indicated that IgA is a multifaceted molecule that can display both pro- and anti-inflammatory properties depending on the environment and can interact with FcαRI on the surface of eosinophils and neutrophils [79,80]. Our data also indicate that plasma from EN failed to significantly inhibit the release of ECP but suppressed histamine and NE suggesting that IgA in the plasma of EN selectively modulate neutrophil and basophil but have less effect on eosinophils.

Our data also indicate that except CP, IgG4 from all clinical groups suppress granulocytes after interaction with both FcγRI and FcγRII confirming results of previous studies indicating that IgG4 binds to FcγRI, FcγRIIa, FcγRIIb and FcγRIIc [81–83]. Activatory FcγRs typically signal through an immunoreceptor tyrosine-based activation motif (ITAM) whereas the inhibitory FcγRIIb triggers signals via immunoreceptor tyrosine-based inhibitory motif (ITIM) [84,85]. Stimulation through ITAM pathway leads to pro-inflammatory activity resulting in destruction and clearance of antigens by phagocytosis, ADCC and promotion of antigen presentation [84]. Bruhns et al. further suggested that IgG4 antibodies display a higher affinity for the inhibitory receptor FcγRIIb [86]. This suggests, in our settings, that IgG4 antibodies may exert their suppressive properties via two distinct but complementary pathways. Suppressive IgG4 antibodies very likely bind to the inhibitory FcγRIIb and deliver a direct anti-inflammatory signal while impeaching pro-inflammatory antibodies (IgG-1-3) to interact with FcγRI.

While investigating the role of immunoglobulins in the modulation of granulocyte activation, we used affinity-purified total IgG, IgA and IgG4. The use of non-antigen specific antibodies was due to technical limitations associated with the amount of patient’s material available. However, the incubation of granulocytes with anti-IgE and IL-3 allows non-antigen specific stimulation of granulocyte subpopulations. Also, due to the well-known technical difficulties associated to the cryopreservation of granulocytes [87,88], the present study used a heterologous system where sera and purified antibody fractions from Ghanaian patients and controls were tested on heterologous granulocytes from healthy European blood donors. Even though a certain level of alloreaction cannot be excluded, our data are validated by the use of the same background for all tested samples. Indeed, all plasma or purified antibody fractions were tested on granulocytes of the same group of donors (n = 9). Also, the use of both heterologous and autologous settings for non-endemic controls showed no impact on the granulocyte activation and histamine release (S6 Fig).

Even though the sample size is relatively small due to the difficulty to recruit patients that have received no anti-filarial treatment after extensive mass drug administration programs and the significant reduction of the disease burden in this region [89,90], the current study extends previous findings suggesting that expression of IgG4 in asymptomatic Mf+ individuals is associated with inhibition of granulocyte functions [10,91,92] and suggests that prominent IgA expression in EN also affects granulocytes’ functions. Our data also provide new insights on a possible role of functional modulation of IgG4 antibodies in CP-patients, providing possible novel clarifications of the mechanisms through which tolerance or pathology is induced in LF, and suggest that IgA and IgG4 may represent meaningful candidates for targeted therapy against LF. Modulating IgG4 and IgA expression and functional properties may for example contribute in the future to the reduction of inflammatory damages in patients with chronic filarial infections.

## Supporting information

S1 Fig Determination of optimal stimulation concentrations. Freshly isolated granulocytes from healthy blood donors (n = 9) were stimulated with increasing concentrations of rIL-3 (0.5 ng/ml, 1 ng/ml, 2 ng/ml, 4 ng/ml and 8 ng/ml) (A) and anti-IgE (3.12 ng/ml, 6.25 ng/ml, 12.5 ng/ml, 25 ng/ml and 50 ng/ml) (B) alone or in combination (C-E). Supernatants were collected after 30 min and 18 hours of incubation at 37°C. The cells were harvested after 18 hours and stained for CD63 and HLADR expression (A-E). The histamine release was assessed in each well per ELISA (F). Activated granulocytes were characterized as CD63+/HLADR- cells. Bars represent means ± SEM of the percentage of activated granulocytes or the levels of histamine. The lowest concentrations that induced the maximal activation and histamine release (2 ng/ml for rIL-3 and 25 ng/ml for anti-IgE) were identified and used in the following experiments. Graphs are representative of 3 independent experiments.
(TIF)

S2 Fig Plasma from EN and Mf+ individuals actively impair granulocyte activation and degranulation. Freshly isolated granulocytes from healthy blood spenders (n = 9) were stained with CD66b, representing granulocyte subtype cells (A). Granulocytes were stimulated with IL-3 (2 ng/ml), anti-IgE (25 ng/ml), and Brugia antigen extracts (10 μg/ml) (B) and then cultured in presence of 5% (v:v) of plasma, containing 5 μg/ml total proteins, of either non-endemic controls (NEC) (C), endemic normal (EN) (D), microfilaria positive (Mf+) (E), microfilaria negative individuals (Mf-) (F) or plasma of chronic pathology patients (CP) (G). After 18 hours of culture, cells were stained and the proportion of activated granulocytes cells characterized as CD63+/HLADR- cells (1 representative dot plot) and the release of histamine in supernatants (H) were determined. Graphs are representative of 3 independent experiments and bars represent means ± SEM. Statistical comparison was based on Kruskal-Wallis one-way ANOVA followed by Dunn post-hoc test. The indicated p-value refers to the significance level among all groups according to Kruskal-Wallis test. Asterisks indicate the level of differences after Dunn’s multiple comparisons test; *: p<0.05; ***: p<0.001.
(TIF)

S3 Fig Purity of IgG fractions and effect on histamine release. IgG molecules were depleted from the plasma of EN, Mf+, Mf- and CP using protein-G based affinity chromatography. Luminex bead-based immunoassay was used to control the purity of IgG positive fractions of EN (A), Mf+ (B), Mf- (C) and CP patietnts(D). Furthermore, western blot analysis was performed on both IgG positive (E) and IgG negative fractions (F) for IgG characteristic bands: the IgG heavy chain (50 kDa), the IgG light chain (25 kDa) and a third band (150 kDa) were detectable in eluates (E) but not in negative fractions (F). The effect of both fractions on activated granulocytes was analyzed and the release of histamine after 18 hours of culture was determined (G). Graphs are representative of 3 independent experiments and bars represent means ± SEM. Statistical comparison was based on Kruskal-Wallis one-way ANOVA followed by Dunn post-hoc test. The indicated p-value refers to the significance level among all groups according to Kruskal-Wallis test. Asterisks indicate the level of differences after Dunn’s multiple comparisons test; *: p<0.05; **: p<0.01; ***: p<0.001.
(TIF)

S4 Fig Depletion of IgG4 antibodies abrogates the suppressive properties of the plasma from LF infected individuals. IgG4 fractions were purified from IgG-enriched fractions of EN, Mf+, Mf- and CP individuals using an IgG4 affinity matrix containing an antibody fragment recognizing human IgG4 and the purity of the fractions was controlled with Luminex bead-based immunoassay (A-D). Then, freshly isolated granulocytes from healthy blood spenders (n = 9) were stimulated with IL-3 (2 ng/ml), anti-IgE (25 ng/ml), and Brugia antigen extracts (10 μg/ml) as control or in presence of 2.5 μg/ml of IgG4+ fractions from EN, Mf+, Mf-, CP and the corresponding IgG4- fractions After 18 hours culture, the percentage of activated CD63+/HLADR- granulocytes was determined. Dot plots (E-I) depict the percentages of activated granulocytes after incubation with IgE/Il-3 alone (E) or in combination with IgG4 from EN (F), IgG4 from Mf+ (G), IgG4 from Mf- (H) or IgG4 purified from CP (I). The release of histamine in the presence of IgG4 positive (J) and negative fractions (K) was measured. In addition, expressions of neutrophil elastase (L) and eosinophil cationic protein (M) were determined in culture supernatants. Graphs are representative of 3 independent experiments and bars represent means ± SEM. Statistical comparison was based on Kruskal-Wallis one-way ANOVA followed by Dunn post-hoc test. The indicated p-value refers to the significance level among all groups according to Kruskal-Wallis test. Asterisks indicate the level of differences after Dunn’s multiple comparisons test; **: p<0.01; ***: p<0.001.
(TIF)

S5 Fig IgG4 from EN, Mf+ and Mf- individuals inhibit neutrophil and basophil activation but not eosinophil’s. Freshly isolated granulocytes from healthy blood donors (n = 9) were stimulated with IL-3 (2 ng/ml), anti-IgE (25 ng/ml) and Brugia antigen extracts (10 μg/ml) as control (dark bars) or in presence of 2.5 μg/ml of IgG4 fractions (grey bars) of EN, Mf+, Mf- and CP for 18 hours. Neutrophil population was gated as CD15+/CD16+ cells (A), basophils as CD203c+/CD123+ cells (B) and eosinophils as CD11b+/Siglec8+ cells (C) from granulocyte population and further analyzed for activation characterized by CD63+/HLADR- expression (A-C). Graphs are representative of 3 independent experiments and bars represent means ± SEM. Statistical comparison was based on Kruskal-Wallis one-way ANOVA followed by Dunn post-hoc test. The indicated p-value refers to the significance level among all groups according to Kruskal-Wallis test. Asterisks indicate the level of differences after Dunn’s multiple comparisons test; *: p<0.05; **: p<0.01; ***: p<0.001.
(TIF)

S6 Fig No inhibition effect of plasma from NEC on autologous and heterologous granulocytes. Freshly isolated granulocytes from healthy blood spenders (n = 9) were stimulated with IL-3 (2 ng/ml), anti-IgE (25 ng/ml) and Brugia antigen extracts (10 μg/ml) as control (dark bars) and then cultured in presence of 5% (v:v) of plasma (containing 5 μg/ml total proteins) of either the same donors (light bars) or different donors (grey bars). The proportion of activated granulocyte cells (CD63+/HLADR- cells) was determined after 18 hours of incubation (A). The release of histamine after 30 min (B), and neutrophil elastase (C) and eosinophil cationic protein (D) after 18 hours, was measured in culture supernatants. Bars represent means ± SEM. Graphs are representative of 3 independent experiments.
(TIF)

S1 Table Villages of origin of patients and controls recruited in the study. Patients and controls used in this work were collected between 2008 and 2010 in 5 different villages of the Ahanta West and Nzema East Districts in Ghana. EN = Endemic normal; Mf+ = Microfilaria positive; Mf- = Microfilaria negative; CP = Chronic pathology.
(DOCX)
