# Supplementary figures and images for: Pathological manifestations in lymphatic filariasis correlate with lack of inhibitory properties of IgG4 antibodies on IgE-activated granulocytes
Source: PLoS Negl Trop Dis. 2017 Jul 24;11(7):e0005777. doi: 10.1371/journal.pntd.0005777 (PMC5542694; doi:10.1371/journal.pntd.0005777)

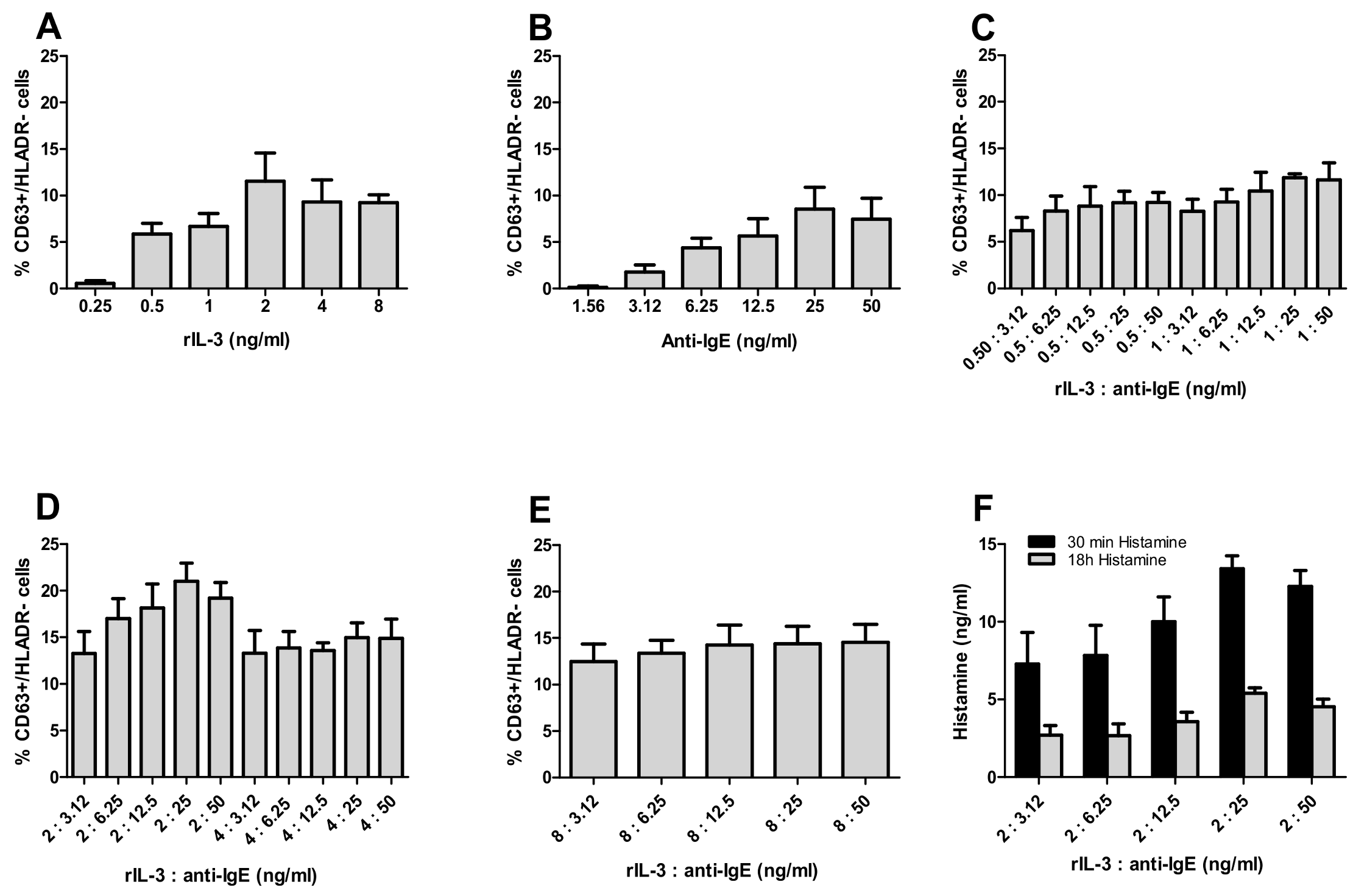

Supplement: S1 Fig — Freshly isolated granulocytes from healthy blood donors (n = 9) were stimulated with increasing concentrations of rIL-3 (0.5 ng/ml, 1 ng/ml, 2 ng/ml, 4 ng/ml and 8 ng/ml) (A) and anti-IgE (3.12 ng/ml, 6.25 ng/ml, 12.5 ng/ml, 25 ng/ml and 50 ng/ml) (B) alone or in combination (C-E). Supernatants were collected after 30 min and 18 hours of incubation at 37°C. The cells were harvested after 18 hours and stained for CD63 and HLADR expression (A-E). The histamine release was assessed in each well per ELISA (F). Activated granulocytes were characterized as CD63+/HLADR- cells. Bars represent means ± SEM of the percentage of activated granulocytes or the levels of histamine. The lowest concentrations that induced the maximal activation and histamine release (2 ng/ml for rIL-3 and 25 ng/ml for anti-IgE) were identified and used in the following experiments. Graphs are representative of 3 independent experiments. (TIF) [file pntd.0005777.s001.tif]

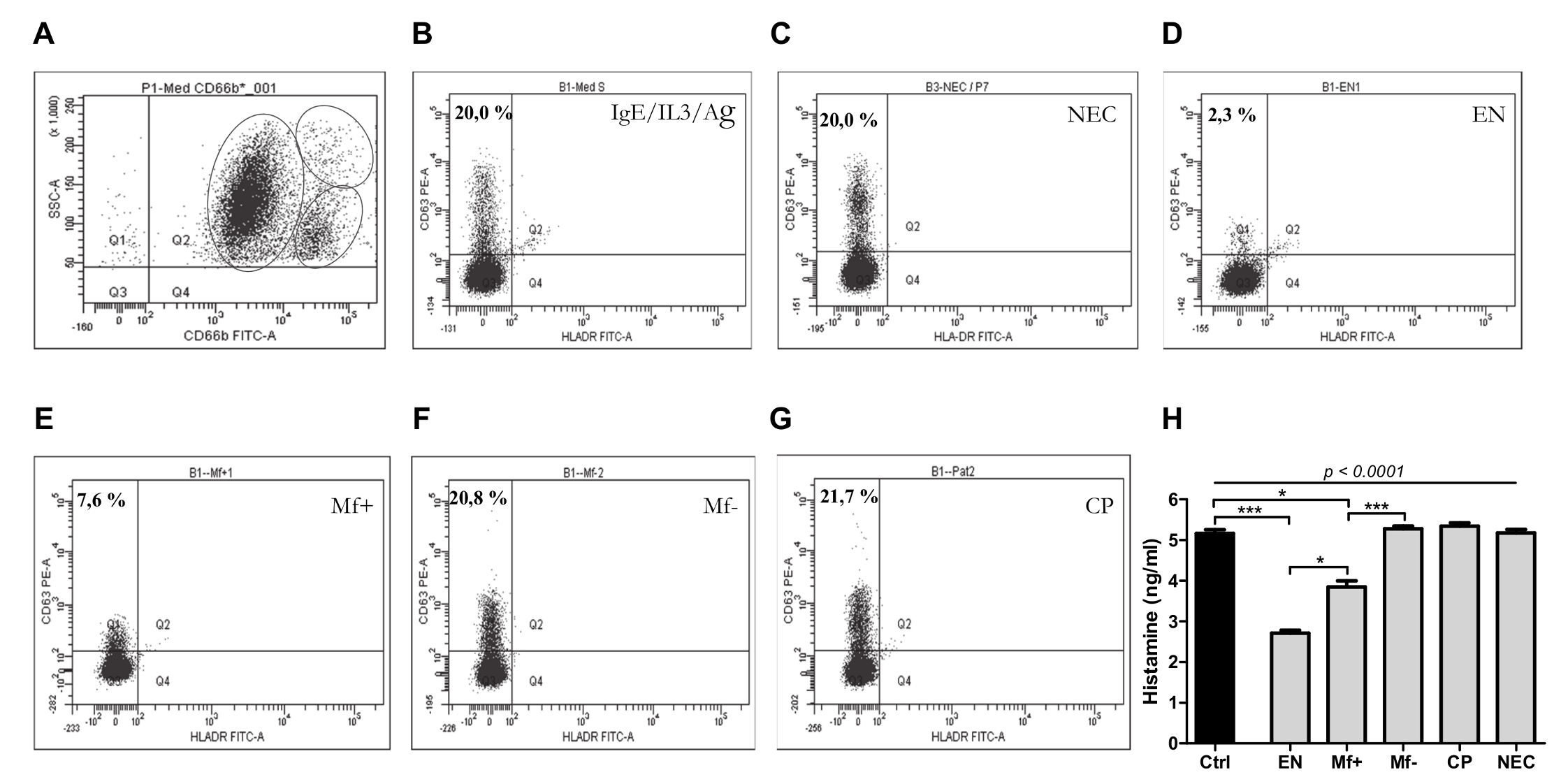

Supplement: S2 Fig — Freshly isolated granulocytes from healthy blood spenders (n = 9) were stained with CD66b, representing granulocyte subtype cells (A). Granulocytes were stimulated with IL-3 (2 ng/ml), anti-IgE (25 ng/ml), and Brugia antigen extracts (10 μg/ml) (B) and then cultured in presence of 5% (v:v) of plasma, containing 5 μg/ml total proteins, of either non-endemic controls (NEC) (C), endemic normal (EN) (D), microfilaria positive (Mf+) (E), microfilaria negative individuals (Mf-) (F) or plasma of chronic pathology patients (CP) (G). After 18 hours of culture, cells were stained and the proportion of activated granulocytes cells characterized as CD63+/HLADR- cells (1 representative dot plot) and the release of histamine in supernatants (H) were determined. Graphs are representative of 3 independent experiments and bars represent means ± SEM. Statistical comparison was based on Kruskal-Wallis one-way ANOVA followed by Dunn post-hoc test. The indicated p-value refers to the significance level among all groups according to Kruskal-Wallis test. Asterisks indicate the level of differences after Dunn’s multiple comparisons test; *: p<0.05; ***: p<0.001. (TIF) [file pntd.0005777.s002.tif]

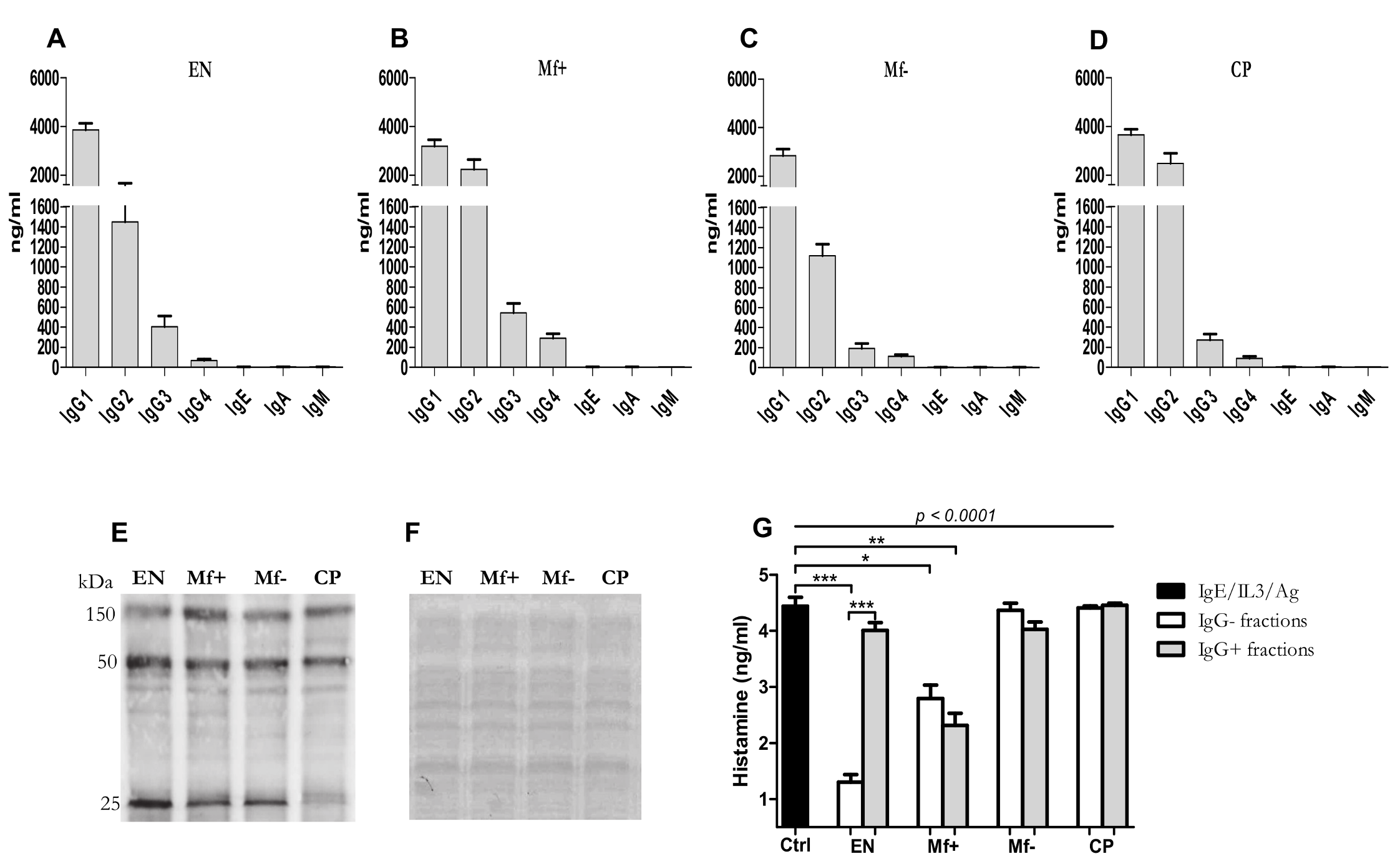

Supplement: S3 Fig — IgG molecules were depleted from the plasma of EN, Mf+, Mf- and CP using protein-G based affinity chromatography. Luminex bead-based immunoassay was used to control the purity of IgG positive fractions of EN (A), Mf+ (B), Mf- (C) and CP patietnts(D). Furthermore, western blot analysis was performed on both IgG positive (E) and IgG negative fractions (F) for IgG characteristic bands: the IgG heavy chain (50 kDa), the IgG light chain (25 kDa) and a third band (150 kDa) were detectable in eluates (E) but not in negative fractions (F). The effect of both fractions on activated granulocytes was analyzed and the release of histamine after 18 hours of culture was determined (G). Graphs are representative of 3 independent experiments and bars represent means ± SEM. Statistical comparison was based on Kruskal-Wallis one-way ANOVA followed by Dunn post-hoc test. The indicated p-value refers to the significance level among all groups according to Kruskal-Wallis test. Asterisks indicate the level of differences after Dunn’s multiple comparisons test; *: p<0.05; **: p<0.01; ***: p<0.001. (TIF) [file pntd.0005777.s003.tif]

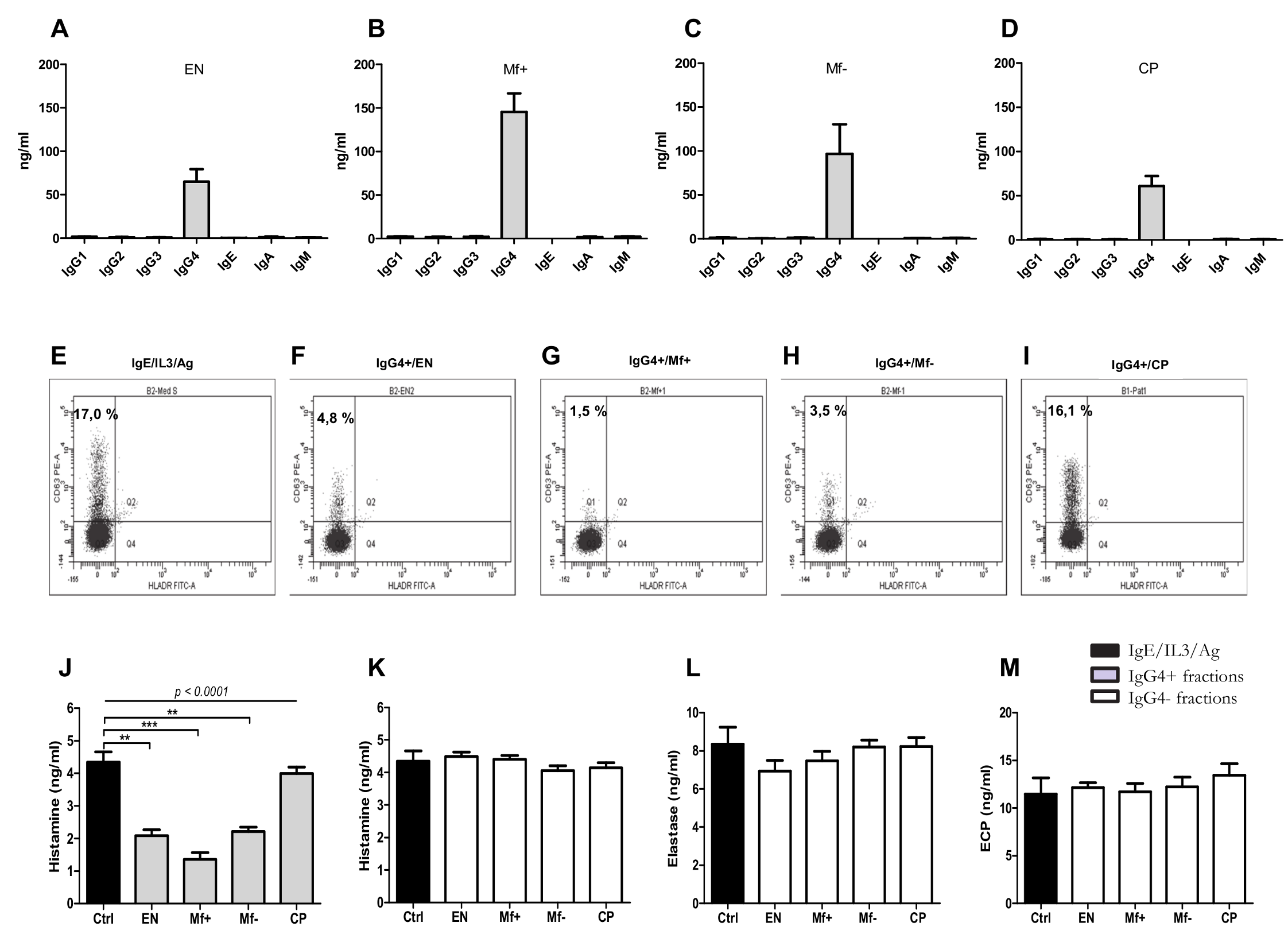

Supplement: S4 Fig — IgG4 fractions were purified from IgG-enriched fractions of EN, Mf+, Mf- and CP individuals using an IgG4 affinity matrix containing an antibody fragment recognizing human IgG4 and the purity of the fractions was controlled with Luminex bead-based immunoassay (A-D). Then, freshly isolated granulocytes from healthy blood spenders (n = 9) were stimulated with IL-3 (2 ng/ml), anti-IgE (25 ng/ml), and Brugia antigen extracts (10 μg/ml) as control or in presence of 2.5 μg/ml of IgG4+ fractions from EN, Mf+, Mf-, CP and the corresponding IgG4- fractions After 18 hours culture, the percentage of activated CD63+/HLADR- granulocytes was determined. Dot plots (E-I) depict the percentages of activated granulocytes after incubation with IgE/Il-3 alone (E) or in combination with IgG4 from EN (F), IgG4 from Mf+ (G), IgG4 from Mf- (H) or IgG4 purified from CP (I). The release of histamine in the presence of IgG4 positive (J) and negative fractions (K) was measured. In addition, expressions of neutrophil elastase (L) and eosinophil cationic protein (M) were determined in culture supernatants. Graphs are representative of 3 independent experiments and bars represent means ± SEM. Statistical comparison was based on Kruskal-Wallis one-way ANOVA followed by Dunn post-hoc test. The indicated p-value refers to the significance level among all groups according to Kruskal-Wallis test. Asterisks indicate the level of differences after Dunn’s multiple comparisons test; **: p<0.01; ***: p<0.001. (TIF) [file pntd.0005777.s004.tif]

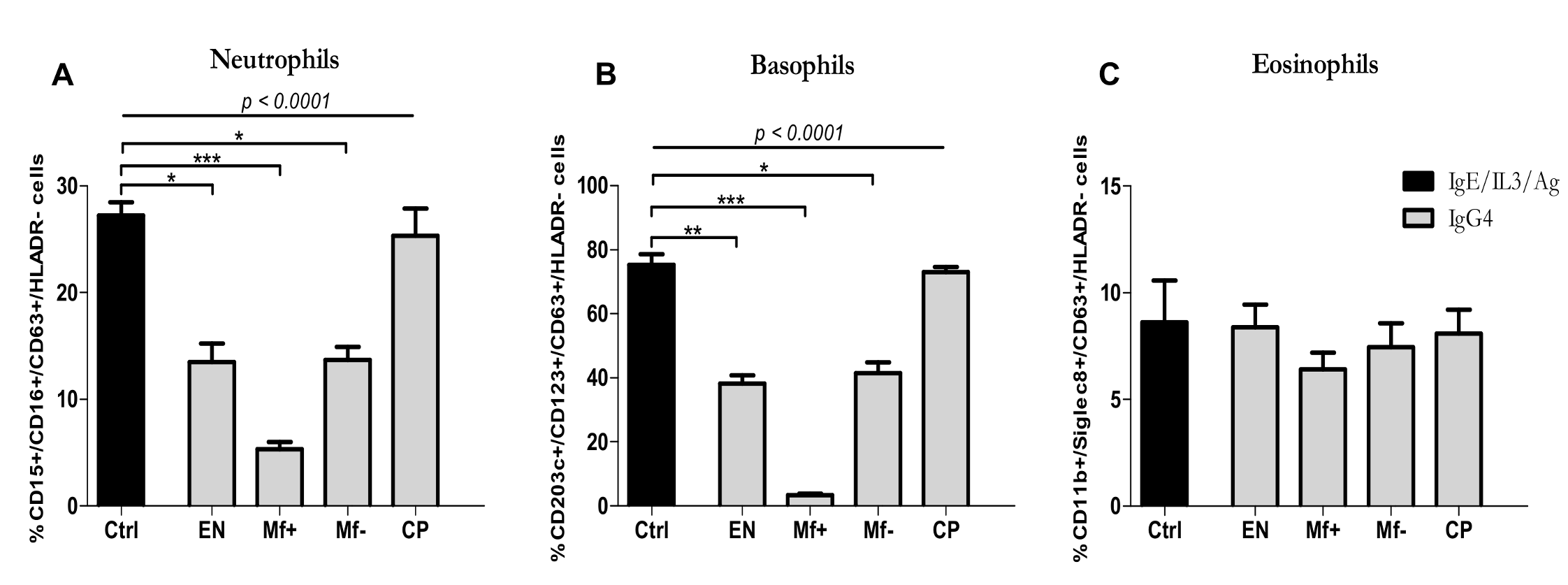

Supplement: S5 Fig — Freshly isolated granulocytes from healthy blood donors (n = 9) were stimulated with IL-3 (2 ng/ml), anti-IgE (25 ng/ml) and Brugia antigen extracts (10 μg/ml) as control (dark bars) or in presence of 2.5 μg/ml of IgG4 fractions (grey bars) of EN, Mf+, Mf- and CP for 18 hours. Neutrophil population was gated as CD15+/CD16+ cells (A), basophils as CD203c+/CD123+ cells (B) and eosinophils as CD11b+/Siglec8+ cells (C) from granulocyte population and further analyzed for activation characterized by CD63+/HLADR- expression (A-C). Graphs are representative of 3 independent experiments and bars represent means ± SEM. Statistical comparison was based on Kruskal-Wallis one-way ANOVA followed by Dunn post-hoc test. The indicated p-value refers to the significance level among all groups according to Kruskal-Wallis test. Asterisks indicate the level of differences after Dunn’s multiple comparisons test; *: p<0.05; **: p<0.01; ***: p<0.001. (TIF) [file pntd.0005777.s005.tif]

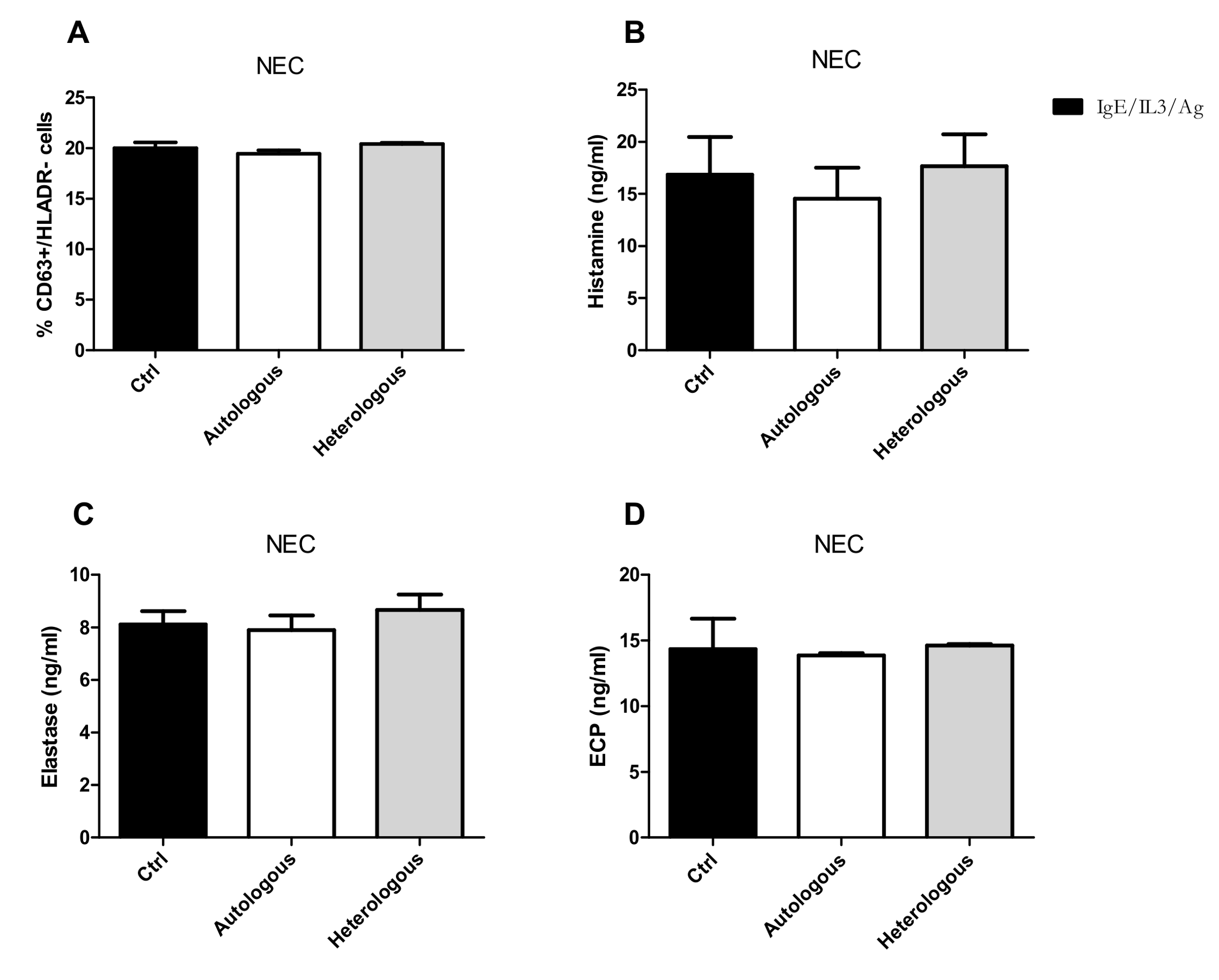

Supplement: S6 Fig — Freshly isolated granulocytes from healthy blood spenders (n = 9) were stimulated with IL-3 (2 ng/ml), anti-IgE (25 ng/ml) and Brugia antigen extracts (10 μg/ml) as control (dark bars) and then cultured in presence of 5% (v:v) of plasma (containing 5 μg/ml total proteins) of either the same donors (light bars) or different donors (grey bars). The proportion of activated granulocyte cells (CD63+/HLADR- cells) was determined after 18 hours of incubation (A). The release of histamine after 30 min (B), and neutrophil elastase (C) and eosinophil cationic protein (D) after 18 hours, was measured in culture supernatants. Bars represent means ± SEM. Graphs are representative of 3 independent experiments. (TIF) [file pntd.0005777.s006.tif]
