# Supplementary material for: Pathological manifestations in lymphatic filariasis correlate with lack of inhibitory properties of IgG4 antibodies on IgE-activated granulocytes
Source: PLoS Negl Trop Dis. 2017 Jul 24;11(7):e0005777. doi: 10.1371/journal.pntd.0005777 (PMC5542694; doi:10.1371/journal.pntd.0005777)
Supplement: S1 Table — Patients and controls used in this work were collected between 2008 and 2010 in 5 different villages of the Ahanta West and Nzema East Districts in Ghana. EN = Endemic normal; Mf+ = Microfilaria positive; Mf- = Microfilaria negative; CP = Chronic pathology. (DOCX) [file pntd.0005777.s007.docx]

**S1 Table. Villages of origin of patients and controls recruited in the study**.

| **Villages** | **Mf+** | **Mf-** | **CP** | **EN** |
| --- | --- | --- | --- | --- |
| Akwanhyiam | 2 | 5 | 4 | - |
| Asamang | 4 | 4 | 5 | 6 |
| Asanta | 3 | 1 | 2 | 4 |
| Agyambra | 3 | 1 | 3 | 4 |
| Mimia | 2 | 3 | - | - |
